# An extensive review of tools for manual annotation of documents

**DOI:** 10.1093/bib/bbz130

**Published:** 2019-12-15

**Authors:** Mariana Neves, Jurica Ševa

**Affiliations:** German Centre for the Protection of Laboratory Animals (BfR), German Federal Institute for Risk Assessment (BfR), Berlin, Germany

**Keywords:** annotation tools, corpus construction, manual annotation

## Abstract

**Motivation:**

Annotation tools are applied to build training and test corpora, which are essential for the development and evaluation of new natural language processing algorithms. Further, annotation tools are also used to extract new information for a particular use case. However, owing to the high number of existing annotation tools, finding the one that best fits particular needs is a demanding task that requires searching the scientific literature followed by installing and trying various tools.

**Methods:**

We searched for annotation tools and selected a subset of them according to five requirements with which they should comply, such as being Web-based or supporting the definition of a schema. We installed the selected tools (when necessary), carried out hands-on experiments and evaluated them using 26 criteria that covered functional and technical aspects. We defined each criterion on three levels of matches and a score for the final evaluation of the tools.

**Results:**

We evaluated 78 tools and selected the following 15 for a detailed evaluation: BioQRator, brat, Catma, Djangology, ezTag, FLAT, LightTag, MAT, MyMiner, PDFAnno, prodigy, tagtog, TextAE, WAT-SL and WebAnno. Full compliance with our 26 criteria ranged from only 9 up to 20 criteria, which demonstrated that some tools are comprehensive and mature enough to be used on most annotation projects. The highest score of 0.81 was obtained by WebAnno (of a maximum value of 1.0).

## Introduction

The use of machine learning algorithms and, more recently, of deep learning algorithms is already a reality in both natural language processing (NLP) and text mining (TM) methods and applications [1]. Such methods usually need to rely on suitable annotated data depending on the problem at hand, whether for document classification [2], named-entity recognition [3] or relation extraction [4]. While some machine learning experiments can rely on unsupervised methods that do not require any previously manually annotated data, supervised (or semi-supervised) learning can achieve higher performance if high-quality annotated data are available.

Manual annotation is the task of reading a particular pre-selected document and providing additional information in the form of the so-called annotations. An annotation can occur at any level of a linguistic component, i.e. document, paragraph, sentence, phrase, word or character. Document-level annotations are useful for supporting document classification tasks, for instance, for identifying documents according to the hallmarks of cancer [2]. Previous work has annotated paragraphs and sentences, for instance, for identifying the various sections of a scientific publication, such as background or methods [5]. Similarly, phrases can be chosen to be annotated, instead of a sentence, to highlight particular expressions that stand for the level of certainty or polarity of findings described in publications [6]. Annotation of words and characters are very common for precisely identifying biomedical entities, such as genes, proteins and diseases [7]. Finally, annotations between linguistic components are also a common task, such as for syntactic dependencies [7] or interactions between drugs [8].

Annotations can occur in different forms depending on the tools in use and/or the goals of the annotation project. It can vary from an unstructured short piece of text, such as a comment on a text passage, as supported by the Hypothesis tool, to structured annotations by means of highlighting text spans or drawing relations between them. The advantage of structured annotations are various, such as their straightforward use for machine learning purposes, the possibility of computing statistics, as well as direct comparison between the various annotators and their agreement. Further, annotations can be either enforced by (strict) predefined guidelines or performed in a relaxed way, without predefined guidelines. The former is always preferable since it enforces homogenity across the various documents and annotators. Adding metadata to annotations is also possible, e.g. normalizing a named entity (e.g. a protein) by precisely mapping it to a particular identifier in a standard database (e.g. UniProt). Finally, the annotation process can be supported by standard terminologies and ontologies, a feature supported by some of the annotation tools (e.g. Knowtator).

Manual annotation is still regarded as the bottleneck for many NLP experiments, given that it is a time-consuming manual process. A new annotation project usually involves a variety of activities [9], such as defining an annotation schema, writing comprehensive annotation guidelines, gathering an adequate document collection, pre-processing these documents according to the task, training experts for the annotation task and building a final consensus corpus. Consequently, the usability and completeness of a tool have a direct impact on the annotation process and can either accelerate or slow it down.

Choosing an adequate annotation tool is a demanding task given the huge number of available tools and the lack of an updated list of annotation tools, along with the advantages and disadvantages of each. Indeed, building a comprehensive list of tools is a challenge because new tools are frequently being released while some of the old ones are no longer maintained. Further, choosing the best suitable annotation tool usually involves trying some of them to obtain insight in their usability and features. This process usually fails to find the executable or source code for the many published tools, as well as failing to install the tools owing to various technical issues. Therefore, any previous review of available tools is welcomed to avoid making poor decisions. Indeed, poor decisions can result in unnecessary waste of time of installing and converting documents to a certain format and later having to migrate them to another tool’s format, and even having to re-train the annotators on another tool.

Because of the reasons stated above, we present a comprehensive review of 78 tools for the manual annotation of documents. We performed a careful search of annotation tools and defined five requirements for the selection of a subset of these for which we performed hands-on experiments based on 26 criteria. As far as we know, this is the most comprehensive review of annotation tools ever performed.

We are aware of previous publications that presented a review of available annotation tools. A recent book described collaborative annotation and included a review of around 20 tools [10]. In addition, we already conducted a comprehensive study on annotation tools that also included hands-on experiments with selected tools [11]. In contrast to our previous study, we now extend the number of analyzed tools (to 78) and list all of the surveyed tools, regardless of whether they meet the five requirements for selection. As expected, many tools have been released since our last survey. Meanwhile, some of the tools selected in the previous survey are now either outdated or no longer available. However, we still cite them for the sake of completeness. From the 15 selected tools that we considered here, only three were included in our previous survey. Our single restriction with regard to our previous survey was the consideration of only Web applications. Distinct from the previous survey, our new evaluation, based on a three-level scale, allowed us to assign a score to each selected tool regarding the fulfillment of the criteria.

Our previous survey was more focused on the biomedical domain, and the selected tools were based on their previous use for the annotation of biomedical corpora or curation, which was rather limiting. We removed this restriction, but we still include criteria related to this field and we address the biomedical domain in a specific subsection in our discussion. Further, whenever possible, our hands-on experiments were based on real use cases using PubMed abstracts.

The next section presents details about our methods, including the five requirements that we defined for the selection of tools and the 26 criteria (each one a three-level scale) under which we evaluated them. Section 3 presents the results of our survey and includes a comprehensive list of the non-selected tools and a detailed evaluation of the selected ones. Finally, we present our discussion regarding various aspects in Section 4.

## Methods

This section describes the methodology used when searching for the annotation tools, the requirements that we defined for selecting a subset of them and the criteria that we considered in the detailed evaluation of the selected ones.

### Initial list of annotation tools

We collected all tools that we were already aware of, including the ones that we considered in our previous survey [11] and those whose publication cites our previous survey.

We made various queries to Google, Google Scholar and PubMed to check all the tools cited in publications related to annotation tools. We noticed that results from Google and Google Scholar can sometimes be slightly different, with Google giving priority for commercial tools and Google Scholar to research tools.

We searched the Corpora mailing list (https://mailman.uib.no/listinfo/corpora) for the past several years as well as recent publications on text classification and information extraction to check how authors have annotated the corpora for their research.

Additionally, we searched the last proceedings (since 2014) of conferences in the field of computational linguistics, such as ACL Anthology (https://aclanthology.coli.uni-saarland.de/) and LREC (http://www.lrec-conf.org/), as well as the proceedings of the BioNLP workshop (https://aclweb.org/aclwiki/BioNLP_Workshop). Unfortunately, many authors do not cite the tool that they used in the annotation process (e.g. [4]) or used a custom (private) tool or an adapted (non-available) version of an existing one [12, 13].

We did not include tools that we would not consider as an annotations tool since they were developed for a particular purpose of the authors, even though they have been used for corpus construction, such as the so-called ‘games with a purpose’ Zombiling [14] and Phrase Detectives [15]. These tools do not allow users to define their own documents nor an annotation schema. We also did not evaluate crown-sourcing tools such as the Amazon Mechanical Turk, since these are out of the scope of this survey. However, application of this tool for linguistic annotation purposes has been discussed in previous work [16, 17].

### Selection of tools

Given the high number of annotation tools that we found, we had to define some requirements with which the tools should comply to be included in our detailed evaluation. These requirements include, among others, features such as being a Web application, supporting a configurable (annotation) schema and whether it is readily available for download (and install) or use. The full set of requirements with which a tool must comply are listed and further explained below:

1. It should be available. [*Available*]
2. It should be a Web application, either as an online or downloadable tool. [*Web-based*]
3. If should be able to be installed in up to 2 h. [*Installable*]
4. It should work for our hands-on experiments. [*Workable*]
5. It should allow for the configuration of a schema. [*Schematic*]

A tool should comply with all the above requirements in order to be selected for further hands-on experiments. The only exception is for tools that are online available, which do not need to comply with the requirement of being installable, since no installation is necessary.

**Fig. 1.**
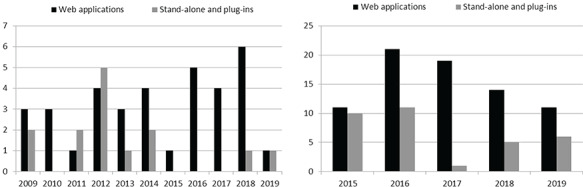
Web-based versus non-Web-based tools: number of annotation tools released in the past 10 years (on the left), and the number of corpora published in the past 5 years (on the right).

**Available.** Our first requirement is that a tool should be readily available, either for direct online use (via a WWW user interface) or for download at the time of writing, without needing to contact the developers. Therefore, we searched for the tool’s URL in its publication (if any) as well as in Google and at the institution’s Web site. This is an indisputable requirement, given that we cannot carry out hands-on experiments on tools that we could not find (e.g. [2]). Unfortunately, many tools had their development discontinued owing to various reasons, such as the end of the project, funding or PhD studies. For the sake of completeness, we list the tools that did not comply with this requirement in case others want to contact the developers.

**Web application.** The tool should be a Web application, i.e. it should be either readily available for online use or downloadable for installation as a Web application. Therefore, we excluded stand-alone systems (e.g. MMAX2 [18]) or plug-ins that run on other tools (e.g. Knowtator [19] runs on Protégé and XConc Suite [20] on Eclipse) or in a browser (e.g. Sapient [21]). Regarding the latter, even though it runs on a browser, it requires the user (annotator) to locally install the tool, just like a stand-alone tool. The Web-based requirement guarantees that the annotators can concentrate solely on the annotation task and do not need to struggle with the installation of the tool. Additionally, other tasks such as schema configuration and document import and export can be carried out by a project leader (if existent), and the annotators can focus on the annotation process. Manual annotation is a demanding and challenging task in itself, and additional tasks might disturb the annotators and compromise the annotation process.

This requirement is supported by two analyses that we carried out: (i) the annotation tools that were published in the past 10 years and (ii) the corpora that was published in the past 5 years, annotated using any of the annotation tools that we found. Figure 1 illustrates the outcomes of these analyses, i.e. the comparison of Web-based on non-Web-based tools that were recently developed or used for corpus construction. Details of the tools included in each of these analyses are presented in the supplementary material. Our analyses clearly show the prevalence of Web applications over stand-alone tools and plug-ins, both when deciding to develop a new tool, as well as when choosing a tool for corpus annotation.

**Installable.** Our third requirement is that a tool, if not available online, should be able to be installed in a maximum of 2 h without needing to contact the developers (Under the assumption of mid-skilled professional.). This is also necessary to enable our hands-on experiments. For the installation, we require that the documentation is comprehensive enough for it to include the tools’ dependencies and instructions on how to start and configure the application. When rejecting a tool under this requirement, we did not consider problems derived from our operating system or other issues related to our servers or environment (e.g. proxy). This requirement was grounds for dismissal only when both authors had issues during the installation process.

**Workable.** Our fourth requirement expects that the tool works properly during our experiments, as this is a prerequisite for our hands-on experiments. Regardless if the tool is locally installed or available online for use, a minimum set of functionalities, defined by our criteria (as presented in Section 2.3), should be available. Therefore, the tool should be intuitive or the documentation comprehensive enough that we did not have to contact the developers for support.

**Schematic.** Our fifth and last requirement states that a tool should allow for the configuration of a schema, i.e. to define labels for the annotation. How this configuration is carried out in the tool is irrelevant, e.g. through the graphical user interface (GUI), command line interface (CLI) or by importing a configuration file. This means that we rejected tools that come with a pre-defined set of labels (e.g. [22]), which do not support this functionality because it was designed for another purpose (e.g. [23]), or that only allow annotation in the form of comments or simple highlighting (e.g. Hypothesis).

### List of evaluation criteria

To evaluate the selected annotation tools, we defined various criteria. We started our list with the criteria that we used in our previous survey [11] and considered which ones were worth keeping and which new ones should not be integrated in this survey. We split our criteria into four categories: (i) publication, (ii) technical, (iii) data and (iv) functional criteria. Each is presented in detail below.

**Publication criteria.** These criteria describe features related to both the tools’ publications and to other publications referencing the use of the tool. These are important aspects to assess the novelty of the tool, as well as its impact on corpus annotation.

- P1 - Year of the last publication;
- P2 - Citations in Google Scholar (as of September 2019);
- P3 - Citations for corpus development (as of September 2019).

Comparing this category to our last survey [11], we changed the criterion that evaluates the number of publications (which describe corpus construction) that we found. We now consider any publication that describes corpus from any domain and not only those from the biomedical domain, which was too restricting.

**Technical criteria.** This group of criteria evaluates technical aspects of the software itself, such as source code availability and the necessity and the easiness of installation (if necessary).

- T1 - Date of the last version (as of August 2019);
- T2 - Availability of the source code;
- T3 - Online availability for use;
- T4 - Easiness of installation;
- T5 - Quality of the documentation;
- T6 - Type of license;
- T7 - Free of charge.

In this group of criteria, we checked various technical criteria of the tools. A tool for which a recent version is available is an important asset and we assess it with the criterion T1. The availability of the source code (criterion T2) allows researchers to customize it according to their needs. Installing a tool is usually a time-consuming task, therefore we check whether it is readily available online (T3) as well as the easiness of installation (T4). A good documentation is an important feature for either installing or properly using a tool, and we evaluate it with the criterion T5. Finally, the type of the license (criterion T6) indicates for which uses a tool is allowed and whether its redistribution is possible.

We evaluated whether a tool is freely available for academia in its full functionality. Nowadays, many implemented tools and algorithms are freely available in open-source repositories. As we also considered commercial tools in the survey, we check whether these are freely available or have a free version that includes most of the important functional features. On one hand, commercial tools come with the uncertainty of whether they will remain freely available in the near future. On the other hand, they might have more functionalities or a more appealing GUI than most of the (freely available) tools developed in academia or research institutes.

We did not consider the criterion ‘type of installation (Web, stand-alone, plug-in)’ from our previous survey [11], given that we selected only Web applications. The same applies for ‘supported operating systems’, given that this is no longer relevant when dealing with Web-based tools.

**Data criteria.** These criteria assess the input and output format of documents, schema and annotations. These were roughly the same criteria that we considered in the previous survey [11].

- D1 - Format of the schema;
- D2 - Input format for documents;
- D3 - Output format for annotations.

The definition of a schema varies across the tools, but it is usually defined either in the GUI or imported from a file. Further, all tools allow importing the documents on which annotation will be performed and exporting the resulting annotations into a file. Criteria D1, D2 and D3 aim to evaluate whether the tools rely on standard formats for importing and exporting these files. Even though using a certain standard, e.g. XML or JSON, is no guarantee of integration of files between various tools, it indicates that researchers can utilize existing programming libraries for parsing and writing the files.

**Functional criteria.** In this group, we evaluated various criteria related to the functionality of the tools.

- F1 - Allowance of multi-label annotations;
- F2 - Allowance of document-level annotations;
- F3 - Support for annotation of relationships;
- F4 - Support for ontologies and terminologies;
- F5 - Support for pre-annotations;
- F6 - Integration with PubMed;
- F7 - Suitability for full texts;
- F8 - Allowance for saving documents partially;
- F9 - Ability to highlight parts of the text;
- F10 - Support for users and teams;
- F11 - Support for inter-annotator agreement (IAA);
- F12 - Data privacy;
- F13 - Support for various languages;

We consider various criteria related to GUI of the tool and how the annotations themselves are carried out in the tool. The ability to support highlighting of text span for the annotations at the levels of sentences, words or characters is evaluated by criterion F9. Further, criterion F1 evaluates whether overlapping text spans are possible, for instance, for assigning more than one label to an annotation. For instance, in the CellFinder corpus [24], the text span ‘mesenchymal precursors’ was annotated as a cell type while the word ‘mesenchymal’ also as an anatomical part.

Annotation of relations between text spans, which is assessed by criterion F3, is an important feature for building corpora for syntactic dependencies or semantic relationships. Further, when annotating long documents, it is essential to choose a tool that is able to display long texts correctly (criterion F7) and that allows saving the annotations partially to later continue the annotation process (criterion F8). We included the new criterion F2 (‘allowance of document-level annotations’), which specifically evaluates the suitability of the tool for this type of annotation, and we included a discussion on this topic (cf. Section 4.6). This is an interesting feature to support corpora construction for document classification.

Besides importing the textual documents on which the annotations will be performed, importing additional resources is also important in some situations. When relying on existing terminologies for the annotation of text, a tool that provides ways to import these resources is an important asset, as indicated by criterion F4. Similarly, in some situations, the user would like to perform the annotation over pre-existing annotations, e.g. to keep the right one and remove the wrong ones, a feature that is evaluated by criterion F5. This feature also evaluates built-in features for automatic predictions and active learning for training a model based on the manual annotations.

Criterion F6 (‘integration with PubMed’) specifically considers an important feature for the biomedical domain, which is the integration with either PubMed ^®^ or PubMed Central ^®^ (PMC). Some annotation tools provide this functionality, such as real-time or off-line integration with PubMed for loading titles, abstracts, meta-data or full texts.

Corpora are usually annotated by more than one expert in order to guarantee the quality of the annotations. Therefore, we evaluate the ability of the tools to support users and teams (criterion F10). After collecting annotations from various experts, a tool that supports a comparison (agreement) between the annotations relieves the researchers of the need to write additional scripts for the post-processing step, as assessed by criterion F11. We include in this criterion various tasks, such as the calculation of simple statistics (inter-annotator agreement) or the support for building a consensus corpus.

The criterion F12 (‘data privacy’) is new and assesses a topic that is important nowadays in which society has concerns about the use of their data by the online applications they use. Further, many research groups are currently annotating sensitive documents such as clinical reports (e.g. [25]). Finally, annotating documents in languages other than English that contain special characters is also an important feature and we assess the suitability of a tool for this task with criterion F13.

Regarding the functional criteria listed in the previous survey [11], we removed those that were no longer relevant, i.e. ‘possibility of using only the keyboard’, ‘automatic selection of a token when clicked’, ‘support for fast annotation’ and ‘allowance of comments on the annotation’. Three other criteria (‘pre-processing the text’, ‘built-in biomedical named-entities recognition’ and ‘easiness of importing pre-annotations’) were somewhat split into the criteria F5 (‘support for pre-annotations’) and D2 (‘input format for documents’).

### Evaluation of tools

We evaluated each selected tool using all of the criteria listed in Section 2.3. For the selected tools that we already considered in our previous survey (i.e. brat, Djangology and MyMiner), we provide a new evaluation of the functional criterion based on the previous survey and our previous hands-on experiments (for the new criteria). However, no new versions of these tools were released since the previous survey. Additionally, we present updated values for the criteria related to the publication metrics or the date of the last version. Our detailed evaluation for each tool is available as supplementary material.

We evaluated each criterion based on a three-level scale: (i) the lowest level means that the criterion was either not covered or only covered in its very basic features, (ii) the medium level means that the criterion was partially covered and (iii) the highest level means that the criterion was fully covered. The application of the three-level scale to each criterion is presented in Table 1. We present these three levels in the form of three colors: ‘gray’ (higher level), ‘light gray’ (medium level) and ‘white’ (lower level).

**Table 1:** Definition of the three-level scale for the evaluation for each criterion

	Total fulfillment (higher level)	Partial fulfillment (medium level)	No fulfillment (lower level)
Criteria			
P1	Last publication since 2009 (past 10 years)	Last publication until 2009 (more than 10 years ago)	No publication found
P2	More than 30 citations	From 11 to 30 citations	Up to 10 citations
P3	More than 10 citations	From 6 to 10 citations	Up to 5 citations
T1	Last version (or commit) since 2014 (past 5 years)	Last version (or commit) until 2014 (more than 5 years ago)	Last version is unknown
T2	Source code available in version control platforms (e.g. GitHub)	Source code available to download	Source code not available
T3	Online system available for use	Online system available with restrictions	Not available online for use
T4	No need to install or easy to install (until half-hour time)	Moderate installation time (from 1- to 2-h time)	Difficult to install (more than 2-h time)
T5	Good documentation (covers most features)	Poor documentation (covers few features)	No documentation
T6	License allows to modify and redistribute the tool	License allows to modify tool	No license available or cannot modify tool
T7	Freely available in its full functionalities	Freely available with limitations	No freely available version
D1	Schema online configurable or uses standard formats (e.g. XML, JSON)	Schema uses non-standard formats	-
D2	Documents based on standard formats (e.g. JSON, XML)	Documents based on non-standard formats	-
D3	Annotations downloaded using standard formats (e.g. JSON, XML)	Annotations based non-standard formats	-
F1	Support for multi-label annotation	-	No support for multi-label annotation
F2	Support for document-level annotation	Poor support for document-level annotation	No support for document-level annotation
F3	Support for relationships	Support for binary relationships or limited support for relationships	No support for relationships
F4	Support for ontologies or terminologies	-	No support for ontologies or terminologies
F5	Active learning from annotated documents	Built-in prediction or upload/import of external annotation	No support for pre-annotations
F6	Integration with Medline/PubMed	Partial support with Medline/PubMed	No integration with Medline/PubMed
F7	Support for full texts	Partial support to full text	No support for full texts
F8	Support for partially saving annotations	Partial support by re-importing annotations	No support for partially saving annotations
F9	Support for text highlighting	-	No support for text highlighting
F10	Users and teams management	Individual user login or restricted user management	No support for users (only anonymous users)
F11	IAA and consensus corpus building	Partial support for either IAA or consensus building	No IAA
F12	Can be used for private data (e.g. medical text)	-	Cannot be used for private data
F13	Support for various languages	Partial support for various languages	No support for various languages

Guidelines for the gray scales when evaluating the tools according to the criteria. The codes for the criteria were defined in Section 2.3.

Not all criteria utilize all three levels of the scale. For some criteria, e.g. ‘suitability for full texts’ (F7), we decided that two levels were enough: either the tool supports it or not. By contrast, we did not define the lowest level for the Data criteria, given that all tools (at least partially) fulfill these features by importing and exporting documents, schema or data in some particular format. Some tools do not support the configuration of an annotation schema (not schematic), but none of our selected tools fit this description, given that this is one of our elimination requirements.

After assessing each criterion, we calculated a final score based on the sum of points obtained by each feature. Fulfilling a criterion (i.e. ‘gray’) corresponds to one point (1.0), partially filling it (i.e. ‘light gray’) to 0.5 point; otherwise, there is no point at all (i.e. ‘white’). Since we did not assign any points to the third level, the latter is ignored for the calculation of the final score. Therefore, the final score is defined by the division of the two following values: (i) the sum of the points obtained from the two highest levels; and (ii) the total number of criteria (e.g. 26 in total).

## Results

We provide the list of all selected and nonselected tools that we found while preparing this survey.

A complete list of all surveyed tools including links to the publications and their Web site, when available, is presented in our GitHub repository.

### Nonselected tools

From the 78 tools that we considered, 63 were not selected for a detailed evaluation of the criteria (cf. Section 2.3). All of the nonselected tools did not comply with at least one of the five requirements that we defined for selecting the tools (cf. Section 2.2). Nevertheless, in this new survey, we decided to provide a comprehensive list of the tools and their elimination grounds for the sake of transparency.

Table 2 summarizes the nonselected tools, the respective publication (whenever available or found) and the reason for exclusion (according to requirements defined in Section 2.2). We cite at least one requirement with which the tools did not comply. However, we did not evaluate all five requirements for all tools since that would require trying to install and use all of them, which is a very time-consuming task. While some requirements can be evaluated by checking the publication (e.g. Available, Web-based and Schematic), some are only possible if the tool is available (e.g. Installable and Workable).

**Table 2:** Summary of the nonselected tools and the respective elimination requirement

Tools	Publications	Elimination requirements	Comments
AGTK	[26, 27]	[Web-based]	Stand-alone tool
AlvisAE	[28]	[Available]	Not available or not found
Anafora	[29]	[Installable]	Documentation still under construction
Analec	[30]	[Web-based]Workable]	Stand-alone tool and documentation only available in French
Annotator	-	[Web-Based][Schematic]	Plug-in, and no configuration of schema
Anotatornia	[31]	[Workable]	Documentation and tool only available in Polish
APLenty	[32]	[Available]	Not available or not found
@Note	[33]	[Web-Based][Schematic]	Stand-alone tool, and no configuration of schema
Argo	[34]	[Workable]	Error when running workflow
Atomic	[35]	[Web-based]	Plug-in in Eclipse
BioAnnotate	[36]	[Web-based]	Stand-alone tool
Bionotate	[37]	[Installable]	No documentation on how to start the system
CCASH	[38]	[Installable]	No documentation on how to start the system
Cadixe	[39]	[Available][Web-based]	Not available or not found, and stand-alone tool
Callisto	[40]	[Web-based]	Stand-alone tool
Cas Editor	-	[Available][Web-based]	Not available or not found, and plug-in in Eclipse
CLARIN-EL	[41]	[Workable]	Log-in did not work
Coco	[42]	[Available][Schematic]	URL does not exist, and not schematic
CRAB reader	[43]	[Available]	Not available or not found, but used in [2]
DOMEO	[44]	[Installable]	No documentation for installation
EasyRef	[45]	[Available][Web-based][Schematic]	Not available or not found, stand-alone tool, and fixed schema
Egas	[46]	[Schematic][Workable]	Schema is limited to some entities and approval of account takes many days
eHost	-	[Available]	Link to the download file does not work
Ellogon	[47]	[Web-based]	Stand-alone tool
EULIA	[48]	[Available]	Not available or not found
GATE Teamware	[49]	[Installable]	Problems working with the various components (Tomcat, MySQL)
GitDox	[50]	[Workable]	Installation worked, but log-in did not
Glozz	[51]	[Web-based]	Stand-alone tool
Hypothesis	-	[Schematic]	No configuration of schema
Inforex	[52, 53]	[Workable]	Documentation only available in Polish
KAFnotator	[54]	[Available][Schematic]	Download file not available, and fixed schema
KCAT	[55]	[Schematic][Web-based]	Entity linking annotation and not Web-based
Knowtator	[19]	[Web-based]	Plug-in in Protégé
MAE	[56]	[Web-based]	Stand-alone tool
Marky	[57]	[Installable]	Documentation is confusing and configuration failed
MDSWriter	[23]	[Schematic]	No configuration of schema
MMAX2	[18]	[Web-based]	Stand-alone tool
NOMAD	[58]	[Web-based]	Stand-alone tool
ODIN	[59]	[Available][Schematic]	Not available or not found, and fixed schema
OLLIE	[60]	[Available]	Not available or not found
PALinkA	[61]	[Available][Web-based]	URL did not work, and stand-alone tool
PACTE	[62]	[Workable]	Documentation not available
PubTator	[22]	[Schematic]	No configuration of schema
Pundit	-	[Schematic]	No configuration of schema
RAD	[63]	[Available]	Not available or not found
SALTO	[64]	[Web-based]	Stand-alone tool
SANTO	[65]	[Workable]	Configuration of schema and document import are confusing
SAPIENT	[21]	[Web-based]	Stand-alone tool
SAWT	[66]	[Available]	Not available or not found
Semantator	[67]	[Available][Web-based]	URL did not work, and plug-in in Protégé
Serengeti	[68]	[Available]	URL does not exist
Slate	[69]	[Available]	Not available or not found
SLATE	[69]	[Web-based]	Run as a terminal
SYNC3	[70]	[Available]	Not available or not found
Textpresso	[71]	[Schematic]	No configuration of schema
UAM Corpus	[72]	[Web-based]	Stand-alone tool
Vogon	-	[Web-based]	Stand-alone tool
WARP-Text	[73]	[Installable]	No documentation for installation
WASA	[74]	[Available]	Not available or not found
WebAnnotator	[75]	[Available]	URL does not exist
WordFreak	[76]	[Web-based]	Stand-alone tool
XConc Suite	[20]	[Web-based]	Plug-in in Eclipse
YEDDA	[77]	[Web-based]	Stand-alone tool

We present the tools by alphabetic order. For more details on the requirements, please refer to subsection 2.2. The URLs of the tools are included in the supplementary material and on-line in our GitHub page.

The URLs of all nonselected tools (when found) are provided as supplementary material and are also available in the GitHub repository. Eight of the 13 tools that we evaluated in our previous survey [11] failed to comply with the Web application requirement and were removed from our new evaluation: @Note, Callisto, GATE, Knowtator, MMAX2, Semantator, WordFreak and XConc Suite. One tool (Bionotate) was not selected because documentation is not detailed enough to instruct the user on how to install and start the application. Further, one tool (Argo) was not selected because an error occurred while executing the workflow and annotating a document was not possible.

We aimed to evaluate at least three requirements for all tools, namely, whether it is available, is a Web application and is schematic. However, we failed to do so in a few situations. We do not know whether eHost and RAD are Web-based or stand-alone tools. Both tools are not available, the publication for the RAD tool is not open access and we did not find a publication for the eHost tool. As for the schematic requirement, the publications from Serengueti and WASA do not provide enough information to evaluate this feature, while we could not access the publication from RAD.

From the 63 nonselected tools, we eliminated tools based on all five requirements. The most frequent elimination reason was the tool not being Web-based (27 times), followed by not available (21 times), not schematic (13 times), not workable (9 times) and not installable (7 times).

The tools that are not a Web application are usually stand-alone tools, but we also found plug-ins to Protégé and Eclipse. We eliminated one tool that runs on a Web browser, namely SAPIENT. It is Web-based but requires installation on the client side and it is not meant to be installed as a Web application and accessed using a browser by various users.

Regarding availability, most of the tools that were eliminated based on this requirement are described in their publications but without a corresponding URL (e.g. CRAB reader). However, some tools had a URL cited in a publication but that no longer existed (e.g. Serengeti).

Seven tools (Anafora, Bionotate, CCASH, DOMEO, GATE Teamware, Marky and WARP-Text) did comply with the above requirements, but their installation failed. The documentation for Anafora, Bionotate, CCASH, DOMEO and WARP-Text is poor and does not provide instructions on how to install or start the application. We did our best to install Marky and even checked a forum in the Web for additional information, but the documentation was too confusing, e.g. making reference to files that we could not find in the installation files. A similar problem occurred with the GATE Teamware, which we decided not to install after experiencing many problems since the tool requires a specific version of Tomcat and MySQL, among other dependencies.

The reasons for a tool being classified as not workable were various, such as not being able to run the workflow that we created owing to an error (Argo), problems related to log-in (CLARIN-EL and Egas), documentation not available in English (Analec and Inforex) or not clear enough to allow for its use (SANTO). The tools that were not schematic were usually those that only allow simple comments and highlighting without a semantic type, such as in the commercial tools Hypothesis and Pundit. Other nonschematic tools include MDSWriter (designed for the annotation of summaries), Textpresso (suitable for data curation) and PubTator (limited to a few biomedical semantic types).

### Selected tools

We list the 15 selected tools, provide a short summary for each and present a detailed evaluation that we carried out based on the 26 criteria (cf. Section 2.3) and the derived scores.

#### List of selected tools

The tools that we selected for a detailed evaluation are listed alphabetically. These tools complied with the five requirements that we defined (cf. Section 2.2). We provide a short summary for each tool that includes whether it is available online, import/export formats, configuration of schema, available tasks and other relevant features. Further details are presented as supplementary material and the selected tools are also listed in our GitHub repository along with links to the publication, when available, and Web site.

BioQRator [78] (http://www.bioqrator.org) This is a tool designed for the annotation of biomedical literature. No local installation is possible, but an online version is available for free. BioQRator supports the BioC format (via file upload) and the retrieval of PubMed articles (via Web services) as input formats, while the export functionality uses the BioC or CSV formats. The annotation schema can be configured in the Web interface by manually adding concepts and assigning them for the annotation of named entities and/or relations. Finally, BioQRator provides pre-annotations based on the Entrez and UniProtKB databases for genes and proteins.

brat [79] (http://brat.nlplab.org) This is one of the most popular tools for the manual annotation of documents and has been used for the development of various corpora (e.g. [80]). brat needs to be locally installed because it is not available online. The schema is configured in a plain text file, and documents are imported in the same format. The annotations can be exported in a similar plain-text format. The highlighting of entities and relations is possible, as well as the normalization to pre-defined terminologies. Although the last version was released in 2012, the tool is still available and is popular in the field. Latest improvements include the embedding of visualizations in HTML pages and integration to external TM tools, among others.

Catma (http://catma.de) This is an online tool that allows for the creation of a corpus collection by importing plain-text documents or the retrieval of HTML documents by entering a certain URL. The user can manually create tagsets and annotate the documents based on these. However, the annotation of relationships is not possible. Automatic annotations are allowed based on queries, e.g. by searching for particular words or terms. Finally, the annotations are exported into the XML format and documents can be shared among users.

Djangology [81] (http://sourceforge.net/projects/djangology) This is a Web-based annotation tool for the collaborative annotation of documents. Users, teams and projects can be configured and managed in the tool as it provides good support for IAA and the construction of a consensus corpus. The annotation schema can be configured in the Web interface, and documents are imported in plain text format. The annotation can be later exported in the plain text format as well.

ezTag [82] (http://eztag.bioqrator.org) This is a tool that allows curators to perform manual annotation and provides training data using the human-in-the-loop process. The tool is available online but can also be locally installed because the source code is available (http://github.com/ncbi-nlp/eztag). ezTag supports both abstracts from PubMed and full-text articles from PMC. It also provides lexicon-based concept tagging as well as state-of-the-art pre-trained taggers, e.g. TaggerOne, GNormPlus and tmVar.

FLAT (http://github.com/proycon/flat) This allows for semantic and linguistic annotations using the FoLiA format [83]. The tool needs to be installed locally as it is not available online. Using the system was not intuitive and the FoLiA XML format, although comprehensive, is not straightforward to use. This is the same format to define new annotation types. We were only able to upload an example document (in Dutch) that was available from the tool but did not succeed in uploading our own biomedical document. FLAT is probably more suitable for linguistic annotations given its support for annotating dependencies, lemmas, chunks, etc.

LightTag (https://www.lighttag.io/) LightTag is an online commercial annotation tool, which is rich in features. No local installation is possible. It supports working in various languages (Arabic, Hebrew and CJK among others), document level, multiword, nested and relationship annotations, among other. Additionally, it uses machine learning to learn from active annotators and suggests possible annotations on unseen text. Annotators can also be split up in teams. Finally, various metrics regarding the entities and annotators are made available, either via the Web interface or via the LightTag API (https://guide.lighttag.io/in-depth/api.html).

MAT (http://mat-annotation.sourceforge.net) This is a tool that includes an active learning functionality. The tool needs to be installed locally because it is not available online. It was not difficult to install, but its use was not intuitive. Although the documentation is comprehensive, it is rather long and some topics are difficult to find, such as the format of the input files. It is possible to configure the schema as an XML import file, including attributes and visualization properties. The annotations can be exported in either JSON or XML formats.

MyMiner [84] (http://myminer.armi.monash.edu.au) This is only available online and cannot be locally installed. Through an integration with PubMed, it is possible to retrieve abstracts and create datasets for later annotation. However, this procedure is rather slow. MyMiner provides support for document labeling, tagging of entity and binary relationships, entity linking and for comparing two annotated files. Pre-annotations can be provided for some entity types based on the Abner and LINNAEUS tools. Documents can be imported in plain-text format and annotations can be exported in a similar format, but the annotation schema can only be configured on-line. No log-in to the system is needed, thus, there is no support for users and teams.

PDFAnno [85] (http://github.com/paperai/pdfanno) This is an open-source tool for the annotation of PDF documents. The documents can only be uploaded in the PDF format, and annotations can be carried out for entities and relationships. PDFAnno also allows for the annotation of nontextual regions in the PDF such as figures and tables. The annotation schema can be configured in the Web interface or imported in the plain text format. Partially saved annotations can be exported in the plain text format and later re-imported for further annotation. Teams and users can only work with this tool if all users have access to the particular folder in the server where all PDFs are stored and where the exported annotations are stored. Annotations from various users can be uploaded simultaneously for the creation of a consensus corpus.

prodigy (https://prodi.gy/) This is a commercial tool, which is freely available only as a demo (showcasing the Web application GUI annotation interface). The developers offer the tool either as a virtual machine (for less tech savvy users) or as local installation. Although the entire setup (installation, starting of the service, setting up initial terminologies for individual labels among other) is done via CLI, the tool is easy to use and made for novice users. It works with multiple file types, which need to be structured appropriately, and it also allows the use of several storage types. prodigy is made with the goal of enabling active learning. As such, it allows for a quick setup of the needed environment and bootstraping the first machine learning models. Currently, it supports the tasks of document classification and named entity recognition. Tasks such as nested/discontinuous spans, relation extraction and automatic linking to knowledge bases/ontologies of interest are currently not supported out of the box. The Web application GUI offers a clean and usable annotation interface and annotations are exported, again via CLI.

tagtog [86] (http://www.tagtog.net) This is an online commercial tool for the annotation of documents on both entity and document levels, as well as of relationships. Because it is a commercial tool, it has some limitations in its free version (e.g. it can only be used online and cannot be installed locally). It provides an easy integration with PubMed for abstracts and full text retrieval and for the definition of a schema in the Web interface. Finally, a machine learning functionality is available for active learning, but we did not evaluate it. The annotations can be linked to some databases (e.g. Entrez and UniProtKB).

TextAE (http://github.com/pubannotation/textae) This allows for the annotation of entities and relationships in documents. It is open source, and it is possible to use the fully functional online version or install it locally. The documents and pre-annotations can be imported in the PubAnnotation JSON format, and the tool provides different views for the annotation of entities and relations. The annotations and documents can be exported in the same JSON file. As an additional feature, it allows for the embedding of annotations in HTML documents for visualization. Finally, it can be used with PubAnnotation [87] for the remote storage of document collections and for integration with PubMed and PMC.

WAT-SL [88] (http://github.com/webis-de/wat) This is a tool developed for segmentation labeling. WAT-SL is not available on-line, and it needs to be locally installed. It is not possible to highlight an arbitrary span of text, and thus the documents must be previously split into the units that will be annotated whether they are paragraphs, sentences, clauses, chunks or tokens. It is simple to install and use, even though the documentation is poor. The schema (segments and labels), input documents and annotations are defined in simple plain-text files. It is possible to define users and assign projects to each of them. A ‘curator interface’ supports the creation of a consensus corpus based on majority voting.

WebAnno (http://webanno.github.io/webanno) [89, 90] This is also a very popular annotation tool that provides full functionality for both semantic and syntax annotations. WebAnno allows for the import of schema and documents and the export of annotations in a variety of formats. The tool works with labels for various syntactic and semantic annotations. WebAnno provides support for users as well as for IAA. It is one of the more comprehensive tools, but its use is not very intuitive (owing to its various annotation layers). However, the documentation is good and covers most of the important topics.

#### Evaluation of selected tools

We present a detailed evaluation of the 15 selected tools based on our 26 criteria (cf. Section 2.3). Each criterion was defined using a three-level scale (cf. Table 1), and the results are depicted in Table 3.

**Table 3:** Visualization of the three-values evaluation of the criteria for the selected tools

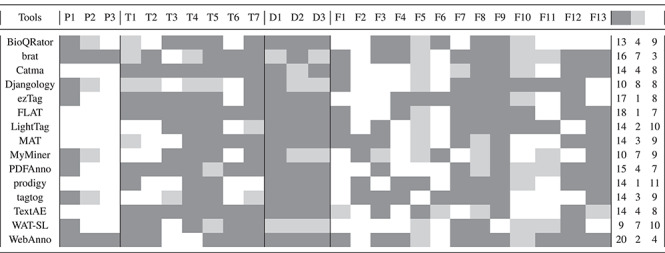

List of criteria: year of last publication (P1), citations in Google Scholar (P2), citations for corpus development (P3), date of last version (T1), availability of source code (T2), online availability for use (T3), easiness of installation (T4), quality of documentation (T5), type of license (T6), free of charge (T7), format of schema (D1), input format for documents (D2), output format for annotations (D3), allowance of multi-label annotations (F1), allowance of document-level annotations (F2), support for relationships (F3), support for ontologies (F4), support for pre-annotations (F5), integration with Medline or PubMed (F6), suitability for full texts (F7), allowance of partial saving (F8), ability to highlight (F9), support for users and teams (F10), support for IAA (F11), data privacy (F12) and support for various languages (F13). For more details on the criteria, please refer to subsection 2.3. The last three columns show the number of occurrences for each value in the three-values scale.

The three tools that fulfilled most of the criteria (gray color) were WebAnno, FLAT and ezTag: 20, 18 and 17 criteria, respectively. One tool fulfilled 16 criteria (brat), one tool 15 criteria (PDFAnno), six tools 14 criteria (Catma, LightTag, MAT, prodigy, tagtog and TextAE) and one tool 13 criteria (BioQRator). Finally, Djangology and MyMiner fulfilled 10 criteria, and one tool (WAT-SL) only nine. Meanwhile, there are three tools (prodigy, LightTag and WAT-SL) that missed the most criteria (11, 10 and 10, respectively), followed by four tools (BioQRator, MAT, MyMiner and tagtog) that missed nine criteria each. The tools that less criteria missed were WebAnno (four criteria) and brat (only three criteria).

WebAnno and brat are the most comprehensive tools and also the most popular tools regarding number of citations.

brat and WebAnno were the only tools that fulfilled all three criteria for publication, while Catma, FLAT, LightTag, MAT, prodigy and TextAE could not even partially fulfill any of these, given that they do not have a corresponding publication. The technical criteria were completely fulfilled by three tools (Catma, FLAT and TextAE), given that they are available online and for download. brat and WebAnno did not score well here because these tools are not available online and are difficult to install. Ten of the 15 tools fulfilled the data criteria, while no tool fulfilled all of the functional criteria, probably owing to the high number of these (13 in total).

**Fig. 2.**
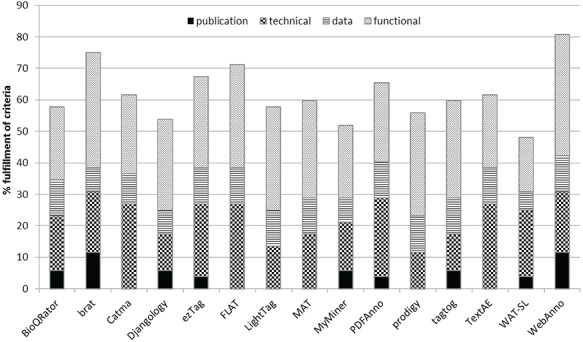
Visualization of the total percentage of fulfillment and the contribution of each criteria category.

The visualization using the three-level scale in Table 3 provides a good overview of the fulfillment. Further, we provide an analysis of the top missing and fulfilled criteria in our discussion section. Based on the results from Table 3, we present in Table 4 the number of points received by each tool per group of criteria and in total, along with the score. We show a visualization of the percentage of fulfillment in Figure 2.

**Table 4:** Summary of the evaluation of all criteria for the selected tools

Tools	P	T	D	F	Total	Scores
BioQRator	1.5	4.5	3.0	6.0	15	0.58
brat	3.0	5.0	2.0	9.5	19.5	0.75
Catma	0.0	7.0	2.5	6.5	16	0.61
Djangology	1.5	3.0	2.0	7.5	14	0.54
ezTag	1.0	6.0	3.0	7.5	17.5	0.67
FLAT	0.0	7.0	3.0	8.5	18.5	0.71
LightTag	0.0	3.5	3.0	8.5	15	0.58
MAT	0.0	4.5	3.0	8.0	15.5	0.60
MyMiner	1.5	4.0	2.0	6.0	13.5	0.52
PDFAnno	1.0	6.5	3.0	6.5	17	0.65
prodigy	0.0	3.0	3.0	8.5	14.5	0.56
tagtog	1.5	3.0	3.0	8.0	15.5	0.60
TextAE	0.0	7.0	3.0	6.0	16	0.61
WAT-SL	1.0	5.5	1.5	4.5	12.5	0.48
WebAnno	3.0	5.0	3.0	10.0	21	0.81
Average	1.0	4.9	2.7	7.4	16.1	0.62
Possible max	3.0	7.0	3.0	13.0	26	1.0

Summary of the points received by each tool for each group of criteria (publication, technical, data and functional), as well as the final total of points and score. The points are calculated according the number of number of gray, light gray and white features as shown in Table 3. In the last two lines, we show the average for each criterion category and the possible maximum number of points and score.

For more details on the criteria, please refer to subsection 2.3.

The scores varied from 0.48 (WAT-SL) to 0.81 (WebAnno), with an average value of 0.62. This shows that many of the tools fulfilled at least half of our criteria. Two tools scored under or slightly over the 0.50 value, namely, WAT-SL (0.48) and MyMiner (0.52). The tools WebAnno (0.81), brat (0.75) and FLAT (0.71) obtained scores over 0.70. Indeed, the average score of all tools is 0.62, which shows an overall reasonable coverage of many criteria by most of the tools. We also calculated a score-per-criteria category to check which categories were less fulfilled by the tools. The group with the most coverage is data (score of 0.88), followed by technical (0.71), functional (0.57) and publication (0.33). Certainly, the reason for the high score of the data category was due to these covering just the two highest levels of fulfillment.

We performed an ablation study to investigate how the scores (and corresponding ranks) of the annotation tools changed by removing each of the groups of criteria separately. On one hand, the ranks for WebAnno and brat varied very little since their positions were always among the top three. On the other hand, the ranks for some tools (Catma, LighTag, PDFAnno, prodigy, tagtog, TextAE) varied substantially depending of the group of criteria that was removed, which indicates that their coverage across the various categories is still unbalanced. Details for the various scores and ranks are presented as supplementary material. In spite of this study, the main goal of the scores was not to decide winners among the tools but to identify those that cover the most criteria that we defined.

## Discussion

This discussion is based on the current state of the art of annotation tools and considers interesting aspects that we observed during this study, such as tools more suitable for the biomedical domain, for the annotation for relationships and the scarce number of tools for document-level annotations.

### State of the art of annotation tools

As we described in the previous section, the average score of 0.62 and minimum score of 0.48 show that all selected tools cover the determined criteria to a great amount. Further, these 15 tools also comply with our five requirements to be in the selected group, especially when considering that 63 other tools could not comply with all requirements.

While the highest scores (up to 0.81) indicate that many of our criteria have indeed been covered by many of the tools, this still leaves almost 20% of the points un-fulfilled (cf. Figure 2). However, WebAnno, brat and FLAT can probably comply with the needs of most users and should be the first choices for those looking for a complete and general-purpose annotation tool. Further, ezTag (fourth best score of 0.67) offers integration with PubMed and PMC, which is particularly important for those working in the field of biomedicine.

Meanwhile, the tools with the lowest scores can also be useful for many researchers, but they are probably more suitable for particular tasks. For instance, WAT-SL, which obtained the lowest score, was designed for segmentation labeling, and thus did not support many of the features that we evaluated. Indeed, this is one of its advantages, as described by the authors [88], over general-purpose annotation tools. The nonselected tools can also be useful for particular tasks, such as Textpresso, which is suitable for the customization of curation tasks [71], Knowtator for annotation based on ontologies [19] and MDSWriter for developing summaries [23].

While many tools have been developed in the past several decades, new tools are constantly being released. Indeed, some of the tools evaluated here were released in 2018 or 2019, e.g. APLenty [32], KCAT [55], PDFAnno [85], SANTO [65], SLATE [69], WARP-Text [64] and WASA [74]. Further, by observing criteria P1 (year of the last publication) and T1 (date of the last version), we conclude that nine of the selected tools were published since 2009, and eight tools released their latest version (or source code’s commit) since 2014.

We hypothesize that the reasons for creating a new tool are various. A particular (but important) feature might be missing; indeed, fulfillment of our criteria is still under 80% for most tools. For instance, PDFAnno was recently created aiming to support the annotation of PDF files, a feature not supported by some of the most popular tools, such as brat. Sometimes, making changes to an existing tool might not be an option because source code is not available for many tools (criterion only fulfilled by eight tools). Further, some tools are developed with a very particular task in mind, as confirmed by the many tools that were eliminated for not being schematic. For instance, MDSWriter is an interesting tool for creating corpora for multi-document summarization.

### Top fulfilled features

When looking at the results in Table 3, no white cells are visible for the data-related criteria. The main reason for this is that our own definition of the levels did not consider the lowest level (white) for this category (cf. Table 1), as already explained in Section 2. Moreover, only 10 (around 22%) of 42 cells (15 tools $\times $ 3 criteria) obtained a partial score. This demonstrates that most of the tools utilize standard input and output formats, such as XML and JSON. In spite of the above, a non-standard format does not necessarily stop users from using a certain tool. For instance, brat is one of the most popular tools, and its publication has more than 400 citations. We found at least 10 citations for corpus construction. However, brat supports no standard formats and uses a plain stand-off text format.

Among the technical criteria, we observe just one white space for the easiness of installation (T4) and for free of charge (T7), and none for the quality of the documentation (T5). The latter indicates that the quality of the documentation of individual tools is either high or adequate. Indeed, tools without a tutorial or documentation were not selected because they were either not installable or not workable. Regarding the easiness of installation (T4), only one tool (WebAnno) was assessed as being difficult to install. Actually, many of the tools that fulfilled this criterion had an online version available (as shown by criterion T3). Finally, only one tool (prodigy) does not have a free-of-charge version of their system.

The top functional criteria fulfilled by most of the tools were support for full text (F7, 11 times, once partially), partially annotating documents (F8, 11 times, 4 times partially), highlighting (F9, 14 tools) and data privacy (F12, 11 times). Indeed, the ability to highlight, save partial annotations and support full text are features that most tools currently provide. Meanwhile, the possibility of data privacy (F12), an important topic nowadays, given the high number of annotations of sensitive documents from patients, is fulfilled by all of the tools except those that are only available online and cannot be locally installed, namely, BioQRator, LightTag, MyMiner and tagtog.

### Top missing features

The criteria related to the publication had the highest percentage of white cells, namely, 28 (around 62%) of 45 cells (15 tools $\times $ 3 criteria). Fifteen of the white cells refer to tools (Catma, FLAT, LighTag, MAT and TextAE) that do not have a publication. Nevertheless, we find scientific publications an important resource for disseminating the tool, describing its main features in a summarized way and presenting a use case. Further, if criterion P1 (year of the last publication) is not met, this has a direct impact on the other two criteria that are associated with the citations even though we found references to some tools’ names in publications. Criterion P1 is also important for tracking future citations to the tool, and thus for enforcing its credibility. The tools that obtained the highest evaluation for all three publication criteria were brat and WebAnno, which are very popular and has already been used for a variety of corpus constructions, also in the biomedical domain [91].

Regarding technical criteria, the top missing features are the type of license (T6, 7 times), online availability of the tool (T3, 6 times), availability of the source code (T2, 6 times, plus 1 partially) and the year of the last version (T1, 3 times, plus 4 partially). More than half of the tools offer a free online version of the tool, thus relieving users from the burden of installation. Further, in spite of the increasing open-source movement, six tools do not provide their code for free, even though three of them are not commercial.

Finally, the most missing functional features include document-level annotation (F2, 11 times), integration with PubMed/PMC (F6, 10 times), the use of ontologies or terminologies (F4, 8 times), IAA (F11, 8 times, plus 4 partially), support for multi-label annotation (F1, 7 times, plus 1 partially), relationship annotation (F3, 6 times, 1 partially) and support for teams and users (F10, 4 times, 8 partially).

We discuss in more detail the support for integration with tools and resources for biomedicine in the next subsection and for document-level annotation in Subsection 4.6.

### Tools suitable for biomedicine

Our survey evaluated whether the tools support the integration of PubMed or PMC (criterion F6), as this facilitates the retrieval, parsing and even pre-processing of documents for further annotations.

Only four of the 15 selected tools fulfilled this functionality, either fully or partially: BioQRator, ezTag, MyMiner and tagtog. Indeed, these are tools that were developed by researchers working on biomedical natural language processing (BioNLP) or TM. ezTag and tagtog are probably the more suitable tools for this purpose because they provide integration with both PubMed and PMC, while BioQRator and MyMiner only provide integration with PubMed. However, the functionality included in tagtog did not always work properly during our experiments (e.g. for the PMIDs 24167564, 24025585 and 23082216). Additionally, two tools (BioQRator and ezTag) support the BioC XML format [92], which is a standard in the BioNLP community. Alternatively, the TextAE tool also allows for integration with PubMed using the PubAnnotation repository.

In spite of the above, the limitations of PubMed and PMC are well known in the biomedical TM community. While Pubmed currently includes more than 29 millions citations (as of August/2019), many of these only contain titles, which are of little use for the researchers. Further, the PMC Open Access subset, which contain full text articles whose license allows automatic processing, currently only contains less than 2.5 million articles (as of August 2019).

Further, some of the tools include pre-trained models or tools for the automatic extraction of semantic entities. We provide a summary of the entity types below:

- BioQRator [78]: genes/proteins;
- ezTag [82]: chemicals, diseases, gene/proteins, species and variations;
- MyMiner [84]: protein, DNA, RNA, cell line, cell type and organism from ABNER [93];
- tagtog [86]: gene/protein.

Regarding the use of general-purpose tools such as brat and WebAnno for the annotation of biomedical documents, these might be more suitable for computational linguists than for biomedical researchers. It is desirable that an annotation tool should be as simple as possible and should not contain any additional functionalities that might disturb its main goal of performing the annotations themselves. In spite of this, many projects have used brat for the annotation of biomedical documents [94, 95]. Finally, we identified some nonselected tools that support integration with either PubMed or PMC, namely, @Note, Argo, Egas, Marky, ODIN, PubTator and Textpresso. Further, Knowtator has also been used for the annotation of biomedical corpora given its good support for ontologies.

### Annotation of relationships

Annotating textual documents with relationships between span texts is a common task when annotating corpora. Relations have been used for the annotation of linguistics elements, e.g. co-references [96] and dependencies [7], as well as semantic relationships between entities, either binaries relations [8] or more complex biological events [95].

From all annotation tools that we reviewed, we found almost 30 available tools that support the annotation of relationships in some way. Among the selected ones, this criterion (F3) is fully supported by BioQRator, brat, MAT, PDFAnno, tagtog, TextAE and WebAnno, while partially supported by MyMiner. We consider that optimal annotation of relationships is performed in a drag-and-drop way, i.e. by graphically drawing arrows (or lines) between entities with the mouse while visualizing the whole text. Indeed, this is already supported by various tools, such as brat, LightTag, PDFAnno, TextAE, XConc Suite and WebAnno. Other tools support this feature using fields in a form or with a table (or matrix), with which the user defines which entities are related, usually only for binary relation but also sometimes by defining a predicate. This is supported by some tools, namely, Anafora, BioQRator, Callisto, Egas, Glozz, Inforex, Knowtator, MAE, MAT, MMAX2, PubTator, SANTO, tagtog, UAM Corpus and WARP-Text. Further, the Vogon tool provides support for annotating the text both on a text-based (in a form) or on graphical way by drawing arrow between boxes (entities). Interestingly, WordFreak present the text in a tree structure and the annotation of relations in a tabular way.

Some tools provide only limited support for this feature. For instance, MyMiner presents a matrix of all annotated entities in which it is possible to annotate binary relations. Being a tool developed for the curation task, Bionotate allows the definition of a curation task that can also be used for annotating a single relationship per snippet of text, as carried out in [97].

### Annotation tools for document classification

The initial goal of this survey was to evaluate tools for annotation on the document level. Owing to the few tools that support this feature (F2), we had to widen the focus of the survey, and thus consider more annotation tools. From Table 3, only four tools address this functionality with span-less annotations: prodigy, MAT, MyMiner and tagtog. Further, we also identified some nonselected tools that seem to support document-level annotation: CRAB reader, LightTag, MAE, Textpresso, UAM Corpus, Vogon and WARP-Text.

MyMiner seems to be the only selected tool that explicitly provides a specific task for this purpose. The user can specify the labels and annotate the documents based on the latter. Given the impossibility of configuring projects and users, the annotators have to define the labels every time a new set of documents is to be annotated. Additionally, the users need to be careful when configuring the labels (use the same names or the same order) in order to be able to later normalize annotations from various annotators or sessions.

Most of the other tools only support this feature by highlighting the text span in the documents. One example involves zero-width annotations or a workaround by highlighting any fixed pre-defined token (e.g. ‘DOCUMENTLABEL’) at the beginning of a document, as suggested by the WebAnno developers (https://github.com/webanno/webanno/issues/923). Using this workaround, tools designed for the annotation of semantic entities and relations can also be used for document-level annotations, but this makes the annotation process more complex and time consuming.

In summary, most tools are far from being suitable for a multi-class, multi-label annotation project. Further, if the use of ontologies is needed, finding an appropriate tool is a challenge given that large terminologies are supported by few tools. Therefore, there is much room for improvement of tools with such features, or even the development of a tool specifically for this purpose.

### Web applications versus stand-alone tools and plug-ins

A total of 18 tools were not selected only based on the requirement that the annotation tools should be a Web application. Figure 1 illustrates the prevalence of the Web applications over the non-Web-based ones well. More specifically, since 2015, only two stand-alone tools have been released, namely, YEDDA [77] and SLATE [69]. The developers of YEDDA highlight the advantages of their tool over the Web applications, e.g. being able to be installed in Windows operating systems. However, this only applies in some particular situations when only Windows computers are available. Further, the developer of SLATE clearly stated that his goal was to build a terminal-based annotation tool that was light, easy-to-install and keyboard-based (private conversation during poster session). However, such a tool is not suitable for annotators who are not familiar with terminals, including many biomedical researchers.

In spite of that, many researchers continue to use annotation tools that are not a Web application (cf. Figure 1). Our analysis showed that the most popular stand-alone (or plug-in) annotation tools are Knowtator, MMAX2 and UAM Corpus. On one hand, Knowtator, which is a plug-in for the Protégé tool, is still popular in the biomedical domain given its good support for ontologies, and was recently used for an extension of the CRAFT corpus [98], as well as in a couple of clinical corpora [99, 100]. On the other hand, MMAX2 was usually used for the annotation of linguistic elements, especially for coreferential and anaphorical relations [101, 102]. Finally, the UAM Corpus tool is usually used for multimodal corpora, given its support for text, images and videos, e.g. in [103].

### Limitations of survey

Even though we surveyed 78 tools, making this the largest analysis of annotation tools to the best knowledge of the authors, our work has some limitations.

Our search of annotation tools included a variety of resources and publications and lasted for many months. We included all tools that we found during this time. However, we are aware that we might have missed some tools, given their seemingly large number. Further, we did not explore tools (apps) developed for mobile devices (if any is available), which can indeed be a desirable feature for annotation while commuting, for instance.

We had to specify some requirements to limit the number of tools with which we could carry out a detailed manual evaluation. Even though we set only five requirements, these resulted in the elimination of 63 tools. However, while some of the requirements might be disputable for some researchers (e.g. Web application, schematic), some of them are mandatory for hands-on experiments (e.g. available, installable, workable).

We defined 26 criteria for the evaluation of the selected tools. While this is an adequate number of features to evaluate the 15 selected tools, we probably missed some other interesting criteria. For instance, some researchers might be interested in features that are specific for domains other than biomedicine or for linguistic tasks (e.g. semantic role labeling or grammatical parsing). We had to dismiss some criteria that, even though important, would require an extremely large amount of time. For instance, we could have evaluated in which browser (e.g. Firefox, Safari or Chrome) or operating system (e.g. Mac OS, Windows or Linux) the tools worked well.

We considered all criteria as being equally important. However, some criteria are certainly more important than others, e.g. support for annotation of relationships (F2) over the year of the publication (P1). Ranking the criteria and assigning higher weights to the more important ones would affect the score (and maybe the rank) of an annotation tool, depending on whether more or less important criteria were fulfilled. Further, we did not consider the inter-dependence among criteria, as it is the case of the publication criteria.

We certainly missed some citations to corpus construction. When searching for corpora that used a particular tool for annotation purposes, we dismissed publications that were not freely available to us (i.e. publications behind a paywall), as we could not check their full text. However, we believe that the main goal of this criterion is to provide an estimate of whether the tool has been (successfully) previously used for corpus construction, rather than a precise number of corpora.

We admit that some of our criteria are rather subjective and that other researchers might have a different opinion than ours for many of them, for example, regarding the quality of the documentation or the easiness of installation. The latter is indeed very dependent on the technical skills of the authors. Nevertheless, we hope that our three-level scale helps users to identify adequate tools for their needs.

Finally, manually reviewing many annotation tools is an exhausting manual process for which few tools are currently available to automatize this task. We did not develop any tool to tackle this problem, but, theoretically, TM is indeed suitable for this task, and is already being used to support construction of systematic reviews [104]. In this new version of the survey, we provide a list of all tools with links to their publications and software, whenever available. We hope that this resource could be the starting point for the development of tools aiming at producing semi-automatic (or even live) surveys.

## Conclusion

We presented the most comprehensive survey available of tools for the manual annotation of textual documents. We reviewed 78 tools, which were evaluated under five requirements that resulted in the selection of 15 tools for detailed hands-on experiments. The evaluation of the selected tools involved 26 criteria defined based on a three-level scale and a final score calculated from the obtained points.

Our results showed that two tools already offer coverage for around 80% of our criteria, while the lowest-scoring and even some unselected tools are still useful for particular curation or annotation tasks. We provided a discussion of the top most frequent and missing features, as well as the suitability of the tools for the biomedical domain and for text classification.

Our review aims to support both users (researchers and annotators) in finding the most suitable tool for their annotation purposes, as well as for developers to identify weaknesses in their (selected or nonselected) tools. In spite of the high number of already available tools, we believe that this study is also a valuable resource for those planning to develop new annotation tools.

##  

**Key points**

- We provided the most recent and comprehensive list of annotation tools for textual documents by reviewing 78 tools.
- We selected 15 tools for hands-on experiments and our results showed that, on average, these tools cover around 60% of the 26 criteria that we defined.
- The top fulfilled features were, among others, the use of standard data formats, easiness of installation or no requirement for installation, good-quality documentation, text span highlighting and support for data privacy.
- The top missing features were, among others, absence of a publication (and consequently, citations), online and source code availability, calculation of an inter-annotation agreement, support for users and teams and integration with PubMed or PMC.
- Four selected annotation tools provide out-of-the box functionality for either named-entity recognition for the biomedical domain or document retrieval from PubMed or PMC.

## Supplementary Material

Supplementary_data_for_the_Survey_annotation_tool_bbz130
