# Supplementary material for: An extensive review of tools for manual annotation of documents
Source: Brief Bioinform. 2019 Dec 15;22(1):146–63. doi: 10.1093/bib/bbz130 (PMC7820865; doi:10.1093/bib/bbz130)
Supplement: Supplementary_data_for_the_Survey_annotation_tool_bbz130 [file supplementary_data_for_the_survey_annotation_tool_bbz130.pdf]

Supplementary material for the publication:

An extensive review of tools for manual annotation of documents

Mariana Neves and Jurica Ševa

Contents

1 Web applications vs. stand-alone tools and plug-ins 3
1.1 Recently developed tools 3
1.2 Recently annotated corpora 3
1.2.1 Web-based 4
1.2.2 Stand-alone 6
1.2.3 Plug-in 7

2 List of criteria 8

3 URLs for nonselected annotation tools 9

4 Detailed evaluation for the selected tools 12
4.1 BioQRator 13
4.2 brat 15
4.3 Catma 17
4.4 Djangology 19
4.5 ezTag 21
4.6 FoLiA Linguistic Annotation Tool (FLAT) 23
4.7 LightTag 25
4.8 MITRE Annotation Tool (MAT) 27
4.9 MyMiner 29
4.10 PDFAnno 32
4.11 prodigy 34
4.12 tagtog 37
4.13 TextAE 40
4.14 WAT-SL 43
4.15 WebAnno 45

5 Details of the scores **47**
5.1 Publication criteria 47
5.2 Technical criteria 47
5.3 Data criteria 48
5.4 Functional criteria 48
5.5 Ablation study 48

1 Web applications vs. stand-alone tools and plug-ins

1.1 Recently developed tools

We made retrieved the annotation tools which were released (published) in the last 10 years, i.e., since 2009, and counted the number of Web-based, stand-alone, and plug-ins, as listed in the table below. The year depicted in the table corresponds to the year of the publication. When creating the Figure 1 included in the manuscript, we merged the stand-alone and plug-in tools into the category of non-Web-based tools.

Year	Web-based	Stand-alone	Plug-in
2009	Anotatornia, Bionotate, Coco	@Note, SAPIENT	-
2010	CCASH, Djangology, KAFnotator	-	-
2011	DOMEO	EasyRef, MAE	-
2012	AlvisAE, brat, CRAB reader, MyMiner	Analec, Glozz, SYNC3	Semantator, WebAnnotator
2013	Anafora, GATE Teamware, PubTator	BioAnnotate	-
2014	BioQRator, Egas, ODIN, tagtog	NOMAD	Atomic
2015	Marky	-	-
2016	CLARIN-EL, MDSWriter, SAWT, WAT-SL, WebAnno	-	-
2017	Argo, GitDOX, Inforex, PACTE	-	-
2018	APLenty, ezTag, PDFAnno, SANTO, WARP-Text, WASA	YEDDA	-
2019	KCAT	SLATE	-

1.2 Recently annotated corpora

We searched for all corpora published in the last five years that used any of the tools that we analyzed in our survey, both selected and non-selected ones. For each annotation tool, we checked in Google Scholar citations to the main publications since 2015. We checked each publication which was available to

us (usually, open access publications), and that did not constitute a book or a dissertation. For annotation tools with many citations, e.g., brat, Knowtator, WebAnno, we restricted our analysis to the first 50 citations as ranked by relevance by Google Scholar. We list all corpora below according to the type of tool (Web-based, stand-alone, or plug-in), to the year of publications, and the tool what was used. When creating the Figure 1 included in the manuscript, we merged the stand-alone and plug-in tools into the category of non-Web-based tools.

1.2.1 Web-based

2015	brat	CADEC corpus [48]<br>drug events in clinical notes [42]<br>adverse drug reactions [83]<br>cyberbullying events [127]
	Coco	paraphrase types [129]
	MAT	Australian historical newspapers [55]
	MyMiner	chemicals and drugs [58]
	tagtog	linked annotations [36]
	WebAnno	Twitter conversations [139]<br>script events [101]<br>supersense tagging for Danish [72]

2016	AlvisAE	Bacteria Biotope corpus [85]
	Anafora	BioCreative/OHNLP corpus [96]<br>SCATE corpus [16]
	brat	SemEval-2016 Task 5 corpus [90]<br>universal dependencies [81]<br>history of Medicine [120]<br>clinical suspicion for Kawasaki disease [31]<br>Sortal anaphora resolution [51]<br>Harvey corpus [108]
	CRAB Reader	cancer hallmarks [12]
	GATE Teamware	social media [18]<br>Arabic machine translation [138]
	Marky	antimicrobial peptide-drug combination networks [44]
	MDSWriter	coherent extracts from heterogeneous sources [15]
	PubTator	chemical-disease relations [66]<br>disease-mutation relations [111]
	Textpresso	genome annotation for P. pastoris [123]<br>functional characterization for C. elegans [19]
	WebAnno	biomedical entity and relation recognition [135]<br>semantic relations for German [109]<br>SemDaX corpus [86]

2017	Anafora	BI-RADS [22]
	brat	argumentation structure in persuasive essays [113]<br>SemEval 2017 Task 10 corpus [6]<br>Swedish clinical corpora [95]<br>clinical trial eligibility criteria [47]<br>factuality values to semantic relations [52]<br>uncertainty to link and rank evidence [141]<br>TwiMed corpus [4]<br>machine translation of error types [30]
	CRAB Reader	cancer hallmarks [11]<br>exposure assessment [64]
	Inforex	Polish and Hebrew corpora, European legal texts [71]
	PubTator	curation for UniProtKB/Swiss-Prot [92]
	Slate	argument structure in Japanese legal documents [133]
	tagtog	mutation mentions [24]
	Textpresso	curation for Echinoidea [59]
	WebAnno	computer-mediated communication [14]<br>narrative texts [77]<br>terminological annotation from online forum [41]

2018	brat	evolution of a scientific field [46]<br>cyberbullying events [126]<br>consumer health questions [50]
	GATE Teamware	causality for Arabic [104]
	MDSWriter	German Diabetes Web Data Set corpus [100]
	PACTE	meeting reports with young offenders [74]
	tagtog	protein localizations [27]
	Textpresso	Gene ontology [78]
	WAT-SL	section annotation of German discharge summaries [70]
	WebAnno	classification of online doctor reviews [98]<br>semantic roles of emotions [53]<br>Slovene user generated content [33]<br>named entity recognition for industrial applications [28]<br>classification of cancer stage [1]

2019	Argo	COPD corpus [45]<br>COPIOUS corpus [121]
	brat	materials science procedural [79]<br>sentence boundary for financial domain [8]<br>chemical compounds from patents [3]
	GitDOX	discourse relations and signaling [69]
	Slate	legal argumentation in Japanese [134]
	Textpresso	curation for Xenopus [80]<br>legal argumentation in Japanese [134]
	WebAnno	multilingual hybrid terms [97]<br>emotional relationships of fictional characters [54]

1.2.2 Stand-alone

2015	MAE	clinical narratives for de-identification [116]<br>risk factors for heart disease in clinical narratives [117]<br>SemEval-2015 Task 8 corpus [93]
	MMAX2	textual phenomena across languages and genres [63]
	SALTO	semantic role labeling for Portuguese [107]
	UAM Corpus	page composition in comics [13]

2016	MAE	conversion of textual clinical diagnostic criteria [43]<br>speculation in policy statements [114]
	MMAX2	anaphoric descriptions [122]<br>coreferences in Wikipedia articles [35]<br>coreference for Basque [112]
	UAM Corpus	text and pictures in multimodal instructions [124]<br>relations in L2 [99]<br>semantic annotation of noun compounds [20]<br>L2 use and misuse of non-numerical quantification [88]

2018	Glozz	spin in biomedical scientific literature [56]
	MMAX2	anaphora resolution [89]<br>coreferential relations in Basque [23]

2019	MMAX2	related works in publications [21]
	SLATE	DSTC7 Task 1 corpus [38]<br>conversation disentanglement [60]
	UAM Corpus	travel narratives [39]<br>videos and photos in Featured Stories of Snapchat [84]<br>pragmatic annotation of speech acts [76]

1.2.3 Plug-in

2015	Knowtator	Arabidopsis leaf growth [119]<br>medication administration errors [67]<br>medication discrepancy [68]<br>prescription in clinical notes [132]

2016	Knowtator	handover information extraction [118]<br>concepts in CRAFT Corpus[10]

2017	Knowtator	anatomical annotation in CRAFT Corpus [9]

2018	Knowtator	quality measures for heart failure [34]<br>errors in dictated clinical documents [142]

2 List of criteria

Category	Code	Description
Publication	P1	Year of the last publication
	P2	Citations in Google Scholar (as of August/2019)
	P3	Citations for corpus development (as of August/2019)
Technical	T1	Date of the last version (as of August/2019)
	T2	Availability of source code
	T3	On-line availability
	T4	Easiness of installation
	T5	Quality of the documentation
	T6	Type of license
Data	D1	Format for schema
	D2	Input format for documents
	D3	Output format for annotations
Functional	F1	Allowance of multi-label annotations
	F2	Allowance of document-level annotations
	F3	Annotation for relationships
	F4	Support for ontologies and terminologies
	F5	Support for pre-annotations
	F6	Integration with Medline and/or PubMed
	F7	Allowance for full texts
	F8	Allowance of saving documents partially
	F9	Ability to highlight parts of the text
	F10	Support for users and teams
	F11	Inter-annotator agreement
	F12	Data privacy
	F13	Support for various languages

3 URLs for nonselected annotation tools

We list below the URLs that we could find for the non-selected tools. However, some of these links might be broken.

Tool	URL
AGTK	http://agtk.sf.net/
AlvisAE	[not found]
Anafora	https://github.com/weitechen/anafora
Analec	http://lattice.cnrs.fr/Telecharger-Analec?lang=fr
Annotator	http://annotatorjs.org/
Anotatornia	http://zil.ipipan.waw.pl/Anotatornia
@Note	http://anote-project.org/
APLenty	[not found]
Argo	http://argo.nactem.ac.uk/
Atomic	http://corpus-tools.org/atomic/
BioAnnotate	http://www.sing-group.org/bioannote/
Bionotate	http://bionotate.sourceforge.net/
Cadixe	[not found]
Callisto	https://mitre.github.io/callisto/
Cas Editor	http://uima.apache.org/d/uimaj-current/tools.html#ugr.tools.ce
CCASH	https://sourceforge.net/projects/ccash/
CLARIN-EL	http://clarin.ellogon.org/
Coco	http://www.lsi.upc.edu/textmess/
CRAB reader	[not found]
DOMEO	https://github.com/domeo/domeo
Egas	https://demo.bmd-software.com/egas/
EULIA	[not found]
eHost	https://code.google.com/archive/p/ehost/
Ellogon	http://www.ellogon.org/
EasyRef	[not found]
GATE Teamware	https://gate.ac.uk/teamware/
GitDox	https://corpling.uis.georgetown.edu/gitdox/
Glozz	http://glozz.free.fr/
Hypothesis	https://web.hypothes.is/
Inforex	https://inforex.clarin-pl.eu/
KAFnotator	[not found]
KCAT	https://github.com/donnyslin/KCAT
Knowtator	http://knowtator.sourceforge.net/
MAE	https://code.google.com/archive/p/mae-annotation/
Marky	http://www.sing-group.org/marky/
MDSWriter	https://github.com/UKPLab/mdswriter
MMAX2	http://mmax2.net/
NOMAD	http://www.ellogon.org/index.php/annotation-tool/nomad-annotation-tool
ODIN	http://www.ontogene.org/odin
PACTE	http://pacte.crim.ca/index_en.html
PALinkA	http://clg.wlv.ac.uk/projects/PALinkA/
prodigy	https://prodi.gy/
PubTator	http://www.ncbi.nlm.nih.gov/CBBresearch/Lu/Demo/PubTator/
Pundit	http://thepund.it/annotator-web-annotation/

Tool	URL
RAD	[not found]
SANTO	https://github.com/ag-sc/SANTO
SAPIENT	http://www.aber.ac.uk/en/cs/research/cb/projects/art/software/
SAWT	[not found]
SALTO	[not found]
Semantator	http://informatics.mayo.edu/CNTRO/index.php/Semantator
Serengeti	[not found]
Slate	[not found]
SLATE	http://jkk.name/slate/
SYNC3	[not found]
Textpresso	http://www.textpresso.org/
UAM Corpus	http://corpustool.com/features.html
Vogon	http://gobtan.sourceforge.net
WARP-Text	https://github.com/venelink/WARP
WASA	[not found]
WebAnnotator	https://addons.mozilla.org/en-US/firefox/addon/webannotator/
WordFreak	http://wordfreak.sourceforge.net/
XConc Suite	http://www.geniaproject.org/tools/xconc
YEDDA	https://github.com/jiesutd/YEDDA

4 Detailed evaluation for the selected tools

4.1 BioQRator

- Institution: National Institutes of Health (NIH), USA
- URL: `http://www.bioqrator.org/`
- Publication: [61]

	Publication
P1	Last publication from 2014, but also one from 2013.
P2	The two publications have a total of 30 citations: 24 (2014), 6 (2013).
P3	We found no corpus development using the tool.

	Technical
T1	Last version was from 2013.
T2	No, source code is not available.
T3	Yes, the tool is available on-line.
T4	There is no need to install the tool since it is available on-line.
T5	No official documentation available, but a step-by-step tutorial is available.
T6	License is unknown
T7	Freely available in its all functionalities

	Data
D1	Schema can be configured on-line (user interface).
D2	Document can be imported as BioC files or PubMed abstracts.
D3	For exporting the annotations, it supports BioC and CSV files.

Functional	
F1	No, multi-label annotations are not possible.
F2	No, document-level annotation is not possible.
F3	Yes, it is possible to create relations between annotations.
F4	Yes, if is possible to import terminologies.
F5	Yes, pre-annotation can be imported.
F6	Yes, there is integration with PubMed for creating the document collection.
F7	No, no allowance for full texts.
F8	Yes, it is possible to save documents partially.
F9	Yes, it is possible to highlight entities.
F10	There is no support for teams, only individual users.
F11	No, there is no inter-annotator agreement.
F12	No, data cannot be private given that it is stored in the tool's servers.
F13	No, multiple languages are not supported.

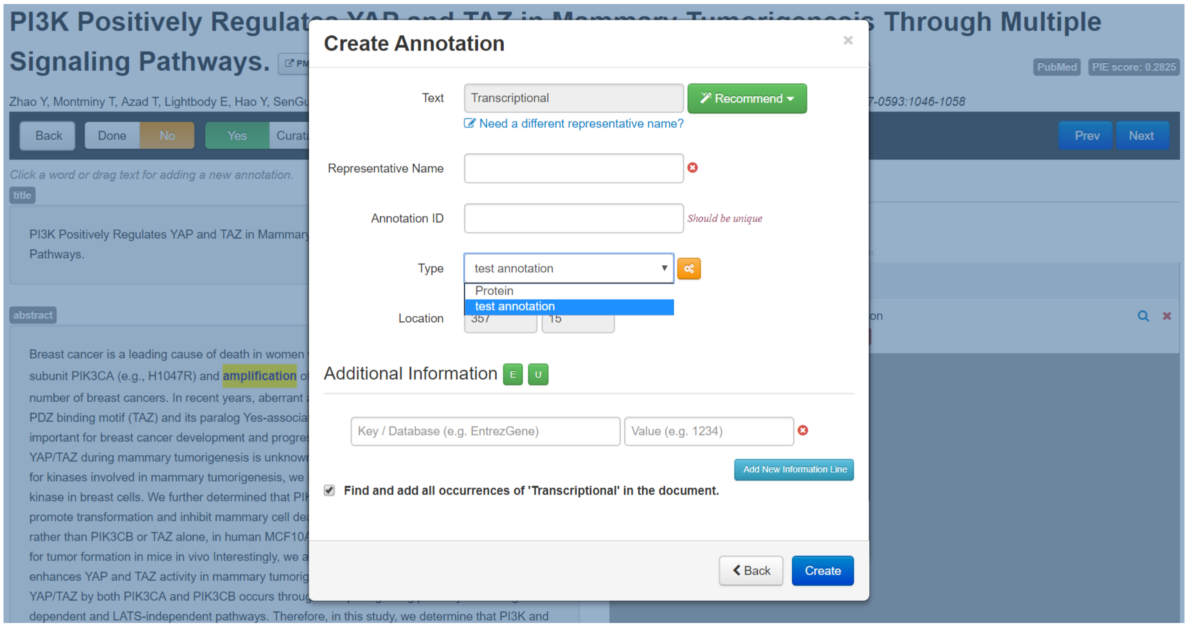

Figure 4.1: Screen-shot of BioQRator.

4.2 brat

- Institution: University of Tokyo (Japan), University of Manchester (UK), Microsoft Research Asia (China).
- URL: `http://brat.nlplab.org/`
- Publication: [115]

Publication	
P1	Publication is from 2012.
P2	The publication has 559 citations.
P3	There are various corpora which were developed using the tool, e.g., [91], [94], [42], [75], [48], [82], [128], [7], [37], [83] and [2].

Technical	
T1	Last stable version (1.3) is from 2012. Last commit in GitHub is at least from 2 years ago.
T2	Yes, source code is available in GitHub.
T3	No, the tool is not on-line available, except for a demo.
T4	It takes some work to install the tool due to its many dependencies.
T5	Documentation is good and clear.
T6	MIT License
T7	Freely available in its all functionalities

Data	
D1	Schema is defined in a non-standard plain text file.
D2	Documents can be imported in plain text format.
D3	Annotations can be exported in a non-standard plain text file.

	Functional
F1	Yes, multi-label annotations are possible.
F2	No, there is no support for document-level annotations.
F3	Yes, it is possible to annotate relations.
F4	Yes, terminologies cannot be imported for normalization.
F5	Yes, external pre-annotations can be imported and pre-annotations can be obtained by integration with other tools.
F6	No, there is no integration with neither Medline nor PubMed.
F7	Yes, full texts can be imported to the tool, even though browsing the documents gets too slow.
F8	Yes, it is possible to save documents partially.
F9	Yes, it is possible to highlight entities.
F10	It is only possible to configure individual users.
F11	No, there is no support for inter-annotator agreement, but it provides the functionality of visualizing two documents side by side.
F12	Yes, data can stay private as a local installation of the tool is need.
F13	Yes, it has been used for other languages, e.g. Japanese

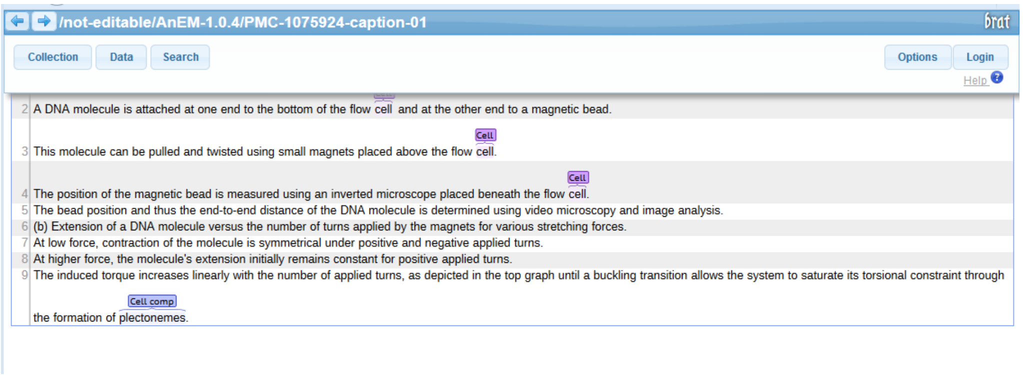

Figure 4.2: Screen-shot of brat.

4.3 Catma

- Institution: University of Hamburg (Germany)
- URL: `http://catma.de/`
- Publication: -

	Publication
P1	We found no publication for the tool.
P2	We found two citations for the tool in Google Scholar.
P3	We found one citation of corpus construction [17]

	Technical
T1	Last commit in GitHub was from a couple of months ago (in 2018).
T2	Yes, the source code is in GitHub.
T3	Yes, the tool is available on-line.
T4	There is no need to install the tool.
T5	Yes, the manual is good and comprehensive.
T6	GNU General Public License, version 3 (GPL-3.0)
T7	Freely available in its all functionalities

	Data
D1	The schema (tagsets) can be configured on-line.
D2	Documents can be imported in the plain text format.
D3	The annotations can be exported in the TEI XML format.

	Functional
F1	Yes, multi-label annotations are allowed.
F2	No, document-level annotations are not allowed.
F3	No, annotation of relationships does not seem to be possible.
F4	No, ontologies and terminologies do not seem to be supported.
F5	Pre-annotations can be automatically tagged based on queries.
F6	No, there is no integration with neither Medline nor PubMed.
F7	Full text can be imported but text is displayed per line and long lines requires using a stroll bar.
F8	Yes, it is possible to save documents partially.
F9	Yes, it is possible to highlight parts of the text.
F10	Yes, documents can be shared among users.
F11	No, inter-annotator agreement is not available.
F12	Yes, data can be private if tool is locally installed. The developers also state that their privacy policy is under the jurisdiction of the EU.
F13	Yes, text in Chinese was correctly displayed and annotated.

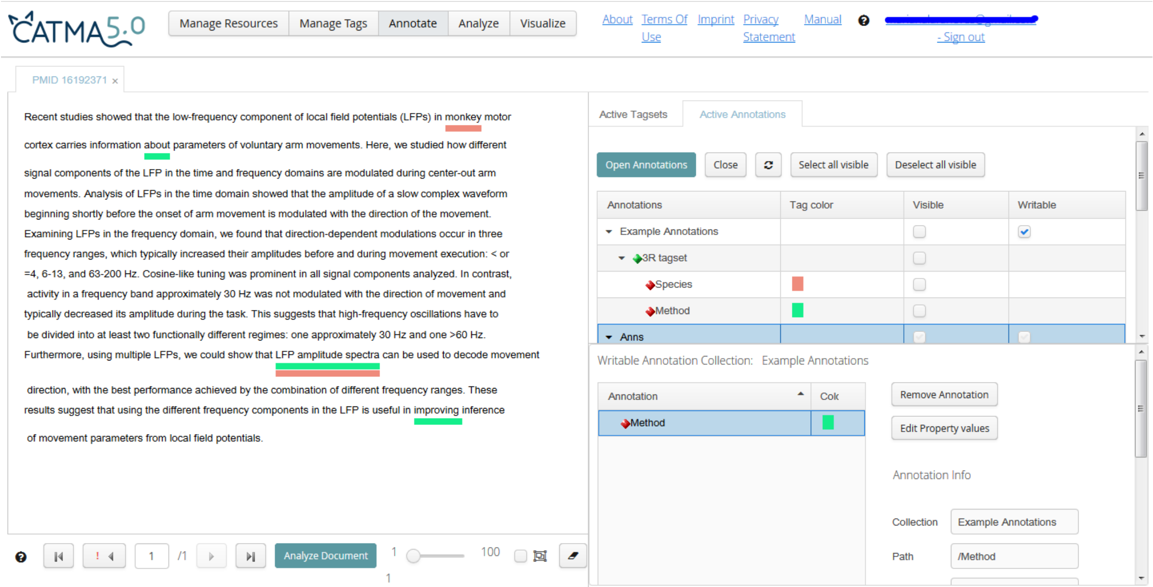

Figure 4.3: Screen-shot of Catma.

4.4 Djangology

- Institution: DePaul University (USA) and Northwestern University (USA)
- URL: `http://sourceforge.net/projects/djangology/`
- Publication: [5]

	Publication
P1	The only publication is from 2010.
P2	The publication has a total of 11 citations.
P3	We did not find any corpus that used the tool but the authors state that they also used it for the BioScope corpus [130].

	Technical
T1	Last version is from 2009.
T2	Yes, the source code is available.
T3	No, the tool is not available on-line.
T4	The tool is a bit hard to install given the need to install an old version of Django. We experienced some problems.
T5	The document is limited to a short wiki.
T6	License is unknown
T7	Freely available in its all functionalities

	Data
D1	The schema can be configured in the GUI.
D2	Documents can be imported in plain text format.
D3	Annotation can be exported to a stand-off plain text format.

Functional	
F1	Yes, multi-label (overlapping) annotations are possible.
F2	No, it does not support document-level annotations.
F3	No, it does not support relationships.
F4	No, there is no support for ontologies nor terminologies.
F5	Yes, pre-annotations can be imported into the database.
F6	No, there is no integration neither with PubMed nor PMC.
F7	Yes, but the text is shown without sentences breaks.
F8	Yes, it is possible to save annotations partially.
F9	Yes, it is possible to highlights entities.
F10	Yes, there is support for teams and projects.
F11	Yes, metrics for IAA can be calculated and a side-by-side comparison is available.
F12	Yes, data can be private given that the tool should be installed locally.
F13	No, multilingual support is not available.

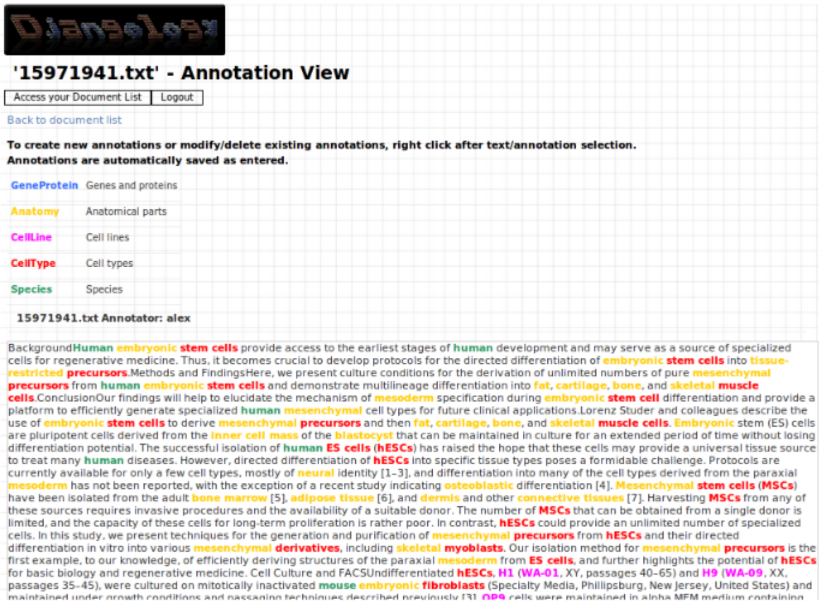

Figure 4.4: Screen-shot of Djangology.

4.5 ezTag

- Institution: National Center for Biotechnology Information (USA)
- URLs: http://eztag.bioqrator.org/, https://github.com/ncbi-nlp/eztag
- Publication: [62]

	Publication
P1	Publication is from 2018.
P2	We found 2 citations for the publication.
P3	No corpus development with the tool were found.

	Technical
T1	Last on-line version seems to be from 2017, but last commit in GitHub was from 2018.
T2	Yes, source code is available.
T3	Yes, the tool is available on-line.
T4	Does not apply, there is no need to install the tool.
T5	Yes, a good documentation (tutorial) is available.
T6	License is unknown
T7	Freely available in its all functionalities

	Data
D1	A schema can be configured on-line or imported as a tab-separated file.
D2	Documents can be imported in the BioC format or as a list of PMID(s)/PMCID(s).
D3	Annotations can be downloaded as a zip file. Individual documents in the BioC format.

Functional	
F1	No, multi-annotations do not seem to be possible.
F2	No, document-level annotation is not possible, unless if using a zero-width annotation.
F3	No, relations cannot be annotated.
F4	Yes, it is possible to upload a lexicon in tab-delimited format.
F5	Yes, pre-annotations are available based on lexicon, training own models with machine learning or from available text mining tools.
F6	Yes, automatic download of documents in a collection through a list of PMID/PMCID values.
F7	Yes, full texts can be imported in BioC format or from PMCIDs.
F8	Yes, it is possible to save documents partially.
F9	Yes, it is possible to highlight entities.
F10	There is no direct support for teams, just for users.
F11	No, there is no support for inter-annotator agreement.
F12	Yes, data can be private if installing the tool locally.
F13	No, multiple languages are not supported.

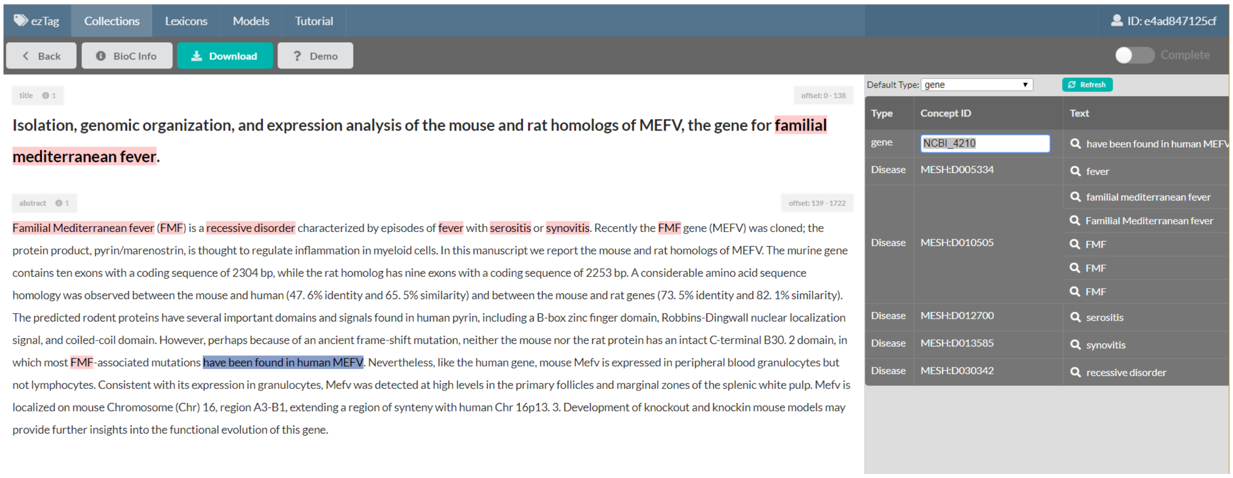

Figure 4.5: Screen-shot of ezTag.

4.6 FoLiA Linguistic Annotation Tool (FLAT)

- Institution: Radboud University Nijmegen (Netherlands)
- URL: `https://github.com/proycon/flat`, `https://flat.science.ru.nl/`
- Publication: -

Publication	
P1	No publication found for the tool, only for the XML format [125]
P2	We found no citations to the tool's URL.
P3	We found no publication describing a corpus development.

Technical	
T1	Last commit in GitHub was on May, 2018.
T2	Yes, source code is available in GitHub.
T3	Yes, the tool is available on-line.
T4	There is no need to install the tool since it is available on-line.
T5	Documentation is good and clear.
T6	GNU General Public License v3.0
T7	Freely available in its all functionalities

Data	
D1	Schema can be defined in the Web interface.
D2	FoLiA XML and CoNNL-U for document input.
D3	FoLiA XML for exporting annotations.

Functional	
F1	Yes, a certain span can have various annotations.
F2	No, document-level annotations should be carried out by highlighting of spans.
F3	No, it is not possible to annotate relationships.
F4	Yes, using the FoLiA XML format.
F5	Yes, by importing the FoLiA XML format.
F6	No, there is no integration with Medline and/or PubMed.
F7	Yes, full texts are allowed.
F8	Yes, it is possible to save documents partially.
F9	Yes, it is possible to highlight parts of the text.
F10	Yes, it is possible to add user and teams using Django admin GUI.
F11	No, inter-annotator agreement is not available.
F12	Yes, data can be kept private as tool need to be installed locally.
F13	Yes, in the FoLiA XML project, an example file in Arabic is included.

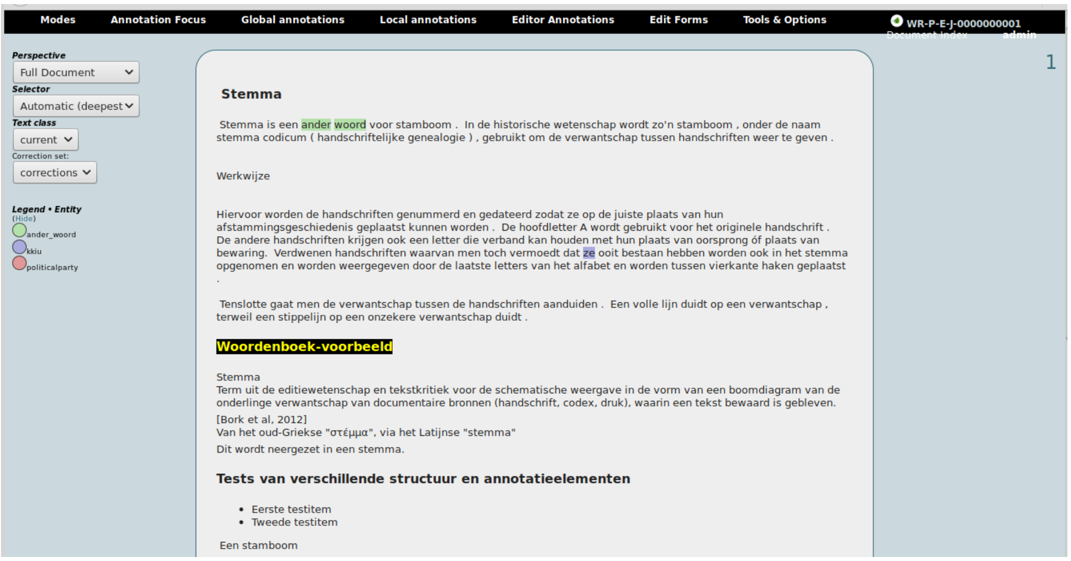

Figure 4.6: Screen-shot of FLAT.

4.7 LightTag

- Company: LightTag
- URL: `https://www.lighttag.io/`
- Publication: -

	Publication
P1	No publication found.
P2	We found no citations to the tool.
P3	We found no publication describing a corpus development.

	Technical
T1	Last version is unknown.
T2	Source code not available.
T3	Yes, the tool is available online.
T4	No installation required.
T5	Documentation is clear, but not complete.
T6	License is unknown.
T7	Freely available with some limitations, e.g., limited number of annotators and annotations

	Data
D1	The schema can be configured on-line in the tool.
D2	The documents can be imported in JSON and CSV.
D3	The annotations can be exported in the JSON format.

	Functional
F1	Yes, multi-label annotations are allowed.
F2	Yes, it supports document-level (span-less) annotations.
F3	Yes, it is possible to annotate relationships.
F4	No, it does not support ontologies and terminologies.
F5	Yes, pre-annotations can be imported.
F6	No, there is no integration neither with Medline nor PubMed.
F7	Yes, full texts are allowed.
F8	Yes, the tool allows saving documents partially.
F9	Yes, it is possible to highlight parts of the text.
F10	Yes, users and teams are supported.
F11	Individual annotator performance metrics, both individually and compared to their peers, is available.
F12	Data is secured under a privacy policy.
F13	Yes (Arabic, Hebrew, CJK among others).

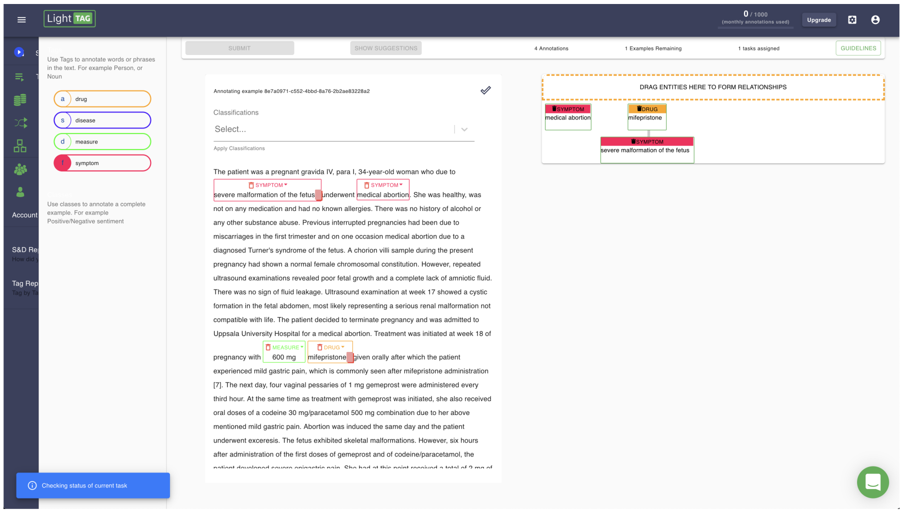

Figure 4.7: Screen-shot of LightTag.

4.8 MITRE Annotation Tool (MAT)

- Institution: The MITRE Corporation
- URL: `http://mat-annotation.sourceforge.net/`
- Publication: -

	Publication
P1	No publication was found.
P2	We found two citations to the tool's URL.
P3	We found one citation for corpus construction, namely, [55].

	Technical
T1	The last version (3.0.5) is from January 28th, 2015.
T2	No, source code does not seem to be available.
T3	No, the system does not have an on-line version available.
T4	Yes, it was easy to install.
T5	The documentation is good, but not very clear and sometimes unnecessarily long.
T6	BSD license
T7	Freely available in its all functionalities

	Data
D1	The schema must be imported from an XML file.
D2	The documents can be imported in free text format.
D3	The annotations can be exported in JSON and XML formats.

	Functional
F1	Yes, multi-label annotations are allowed.
F2	Yes, it supports document-level (span-less) annotations.
F3	Yes, it is possible to annotate relationships using span-less annotations.
F4	The GUI is probably not suitable for a long list of terms from a ontology and terminology
F5	Yes, pre-annotations can be imported.
F6	No, there is no integration neither with Medline or PubMed.
F7	Yes, it is possible to upload full texts and break lines are supported.
F8	Yes, the tool allows saving documents partially by exporting results and importing these back later.
F9	Yes, it is possible to highlight parts of the text.
F10	No, it does not support users nor teams.
F11	No, there is no inter-annotator agreement.
F12	Yes, data can be kept private because the tool needs to installed locally.
F13	Yes, multiple languages are presented, tokenized and possible to annotate.

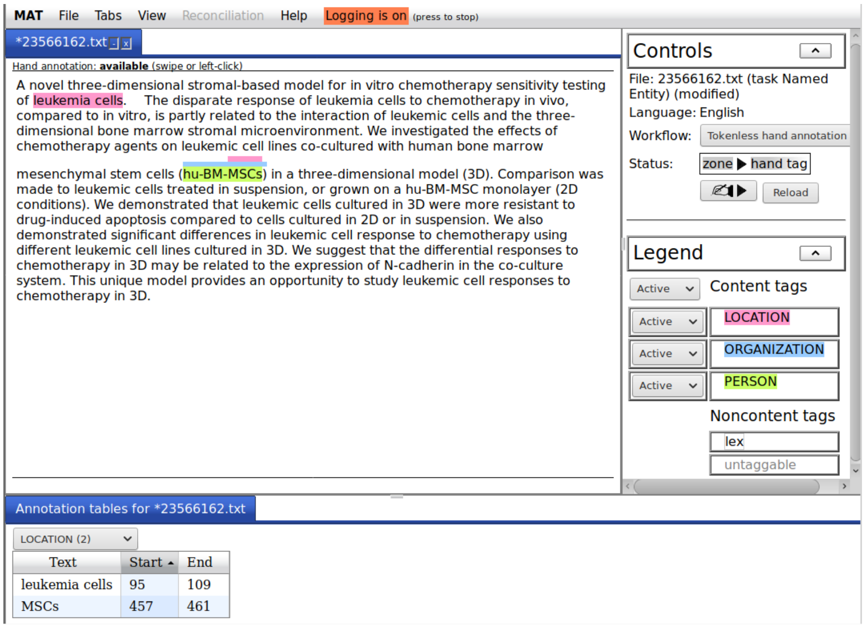

Figure 4.8: Screen-shot of MAT.

4.9 MyMiner

- Institutions: Monash University (Australia), Spanish National Cancer Research Centre (Spain), CNRS (France), CNRS/UI/UII (France) and Indian Institute of Technology (India).
- URL: `http://myminer.armi.monash.edu.au/`
- Publication: [106]

	Publication
P1	The publication is from 2012.
P2	There are 29 citations to the publication (as of August/2018)
P3	We found 2 corpora development using the tool (as of August/2018), namely, [57] and [65], from the developers of the tool.

	Technical
T1	The last version of the tool is unknown.
T2	The source code is not available.
T3	Yes, it is available on-line.
T4	There is no need to install.
T5	The tutorial is short but covers most important aspects.
T6	License is unknown
T7	Freely available in its all functionalities

	Data
D1	The schema can be configures on-line in the tool.
D2	Documents can be imported in a specific plain text format.
D3	The annotations can be exported in a specific plain text format.

Functional	
F1	There is restricted support for overlapping annotations and no support for multi-label annotations.
F2	Yes, there is a specific functionality (document labeling) for document-level annotations.
F3	It is only possible to annotate binary relations.
F4	No, there is no support for terminologies for the annotation itself, only with OBO ontologies for entity linking.
F5	Yes, performed by a couple of text mining tools.
F6	Yes, there is integration with PubMed, but it is very slow.
F7	No, the tool is not suitable for full texts.
F8	Yes, it is possible to save documents partially by exporting and re-importing them.
F9	Yes, it possible to highlight parts of the text.
F10	No, there is no support for neither users nor teams.
F11	No, there is no calculation of IAA, but there is a functionality for comparing files.
F12	No, data cannot be private as the tool is only available on-line.
F13	Yes, text in Chinese could be correctly displayed and annotated.

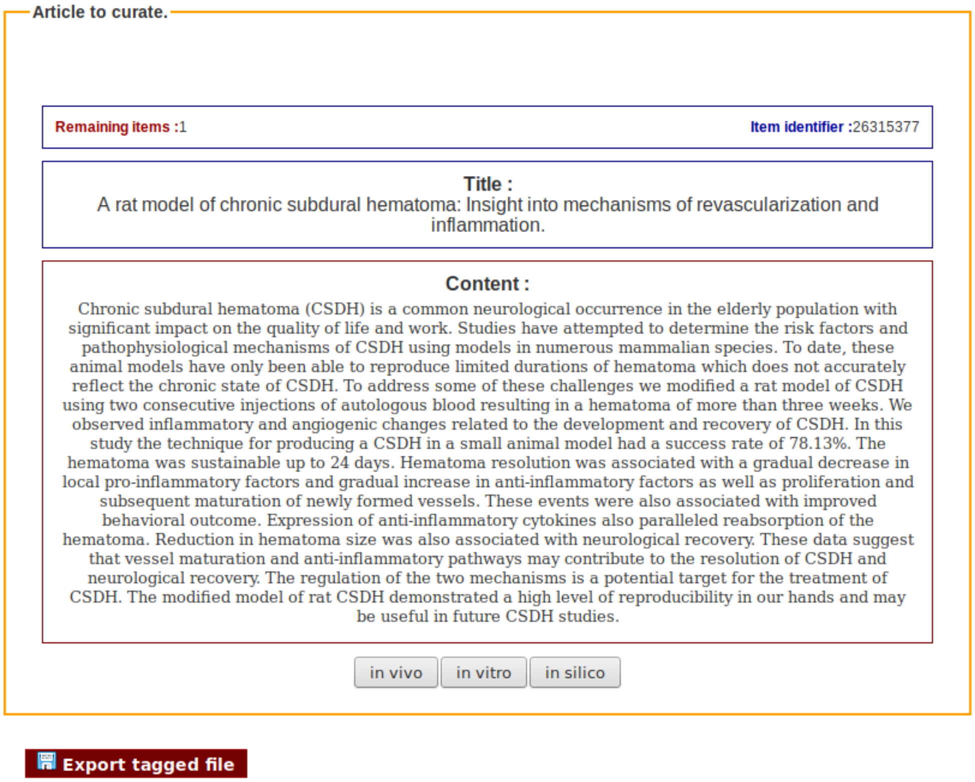

Figure 4.9: Screen-shot of MyMiner.

4.10 PDFAnno

- Institution: Nara Institute of Science and Technology (Japan)
- URL: `https://github.com/paperai/pdfanno`
- Publication: [?]

	Publication
P1	Publication is from 2018.
P2	We found no citations for the publication (as of August/2018).
P3	We found no citations for corpus development (as of August/2018).

	Technical
T1	Last version is 0.4.1 (date unknown) and last commits in GitHub are from July/2018 (as of August/2018).
T2	Yes, source code is available in GitHub.
T3	Yes, the tool is available on-line.
T4	There is no need to install the tool.
T5	Document is poor (README page in GitHub).
T6	MIT License
T7	Freely available in its all functionalities

	Data
D1	The schema can be configured in the tool or imported as a plain text file.
D2	Documents can be imported in the PDF format.
D3	Annotations are exported as plain text files (TOML format [1]).

	Functional
F1	No, multi-label annotations are not possible, only overlapping ones.
F2	No, document-level annotations do not seem to be possible.
F3	Yes, it is possible to annotate relationships.
F4	No, ontologies and terminologies are not supported.
F5	No, pre-annotations do not seem to be supported.
F6	No, there is no integration with Medline and/or PubMed.
F7	Yes, full texts are supported when in the PDF format.
F8	Yes, by exporting the annotations and later re-importing them.
F9	Yes, it is possible to highlight entities.
F10	No, there is no support for user or team management.
F11	It is possible to build a consensus corpus by importing annotations from multiple annotators.
F12	Yes, data can be private if tool is installed locally.
F13	Yes, since the tool relies on PDF files.

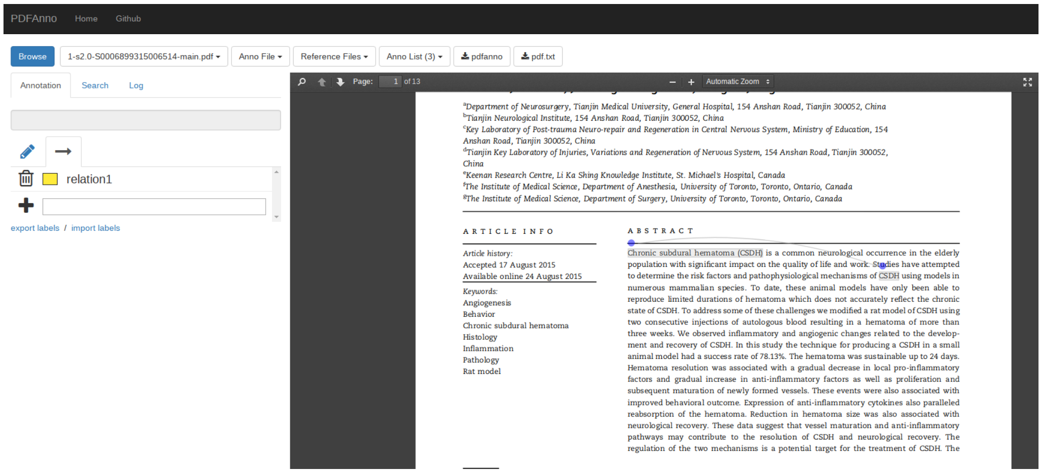

Figure 4.10: Screen-shot of PDFAnno.

4.11 prodigy

- Institution: ExplosionAI GmbH
- URL: `https://prodi.gy/`
- Publication: -

Publication	
P1	No publication available.
P2	No citations found.
P3	No citations for corpora found.

Technical	
T1	Last version from 2019
T2	As this is a commercial tool, no source code is available.
T3	No, the tool is not on-line available, except for a demo.
T4	The tool is easily installed (via pip).
T5	Documentation is extensive and clear, with a lot of examples.
T6	No license information provided.
T7	No freely available version

Data	
D1	Schema is defined in a non-standard command-line interface or .JSONL file.
D2	The tool supports numerous file formats, which need to be structured prior to using.
D3	Yes, the tool offers the possibility to export the annotations as JSON files.

	Functional
F1	No, multi-label annotations are not possible.
F2	Yes, document-level annotations are supported (as a classification task).
F3	No, relation annotations are not supported.
F4	No, importing of ontologies/terminologies is not supported. Bootstrapping a terminology list with word vectors and seeds terms is.
F5	Yes, pre-annotations are supported (and beneficial due to built in active learning capabilities).
F6	No, integration with Medline and/or PubMed Central is not supported.
F7	Yes, full texts are allowed.
F8	Yes, annotators can save partial work.
F9	Yes, annotated parts are visually highlighted.
F10	Teams are not supported.
F11	No automatic inter-annotator agreement is calculated.
F12	Yes, data can be private if tool is locally installed.
F13	Yes, the tools displays non-English (e.g. Chinese) symbols.

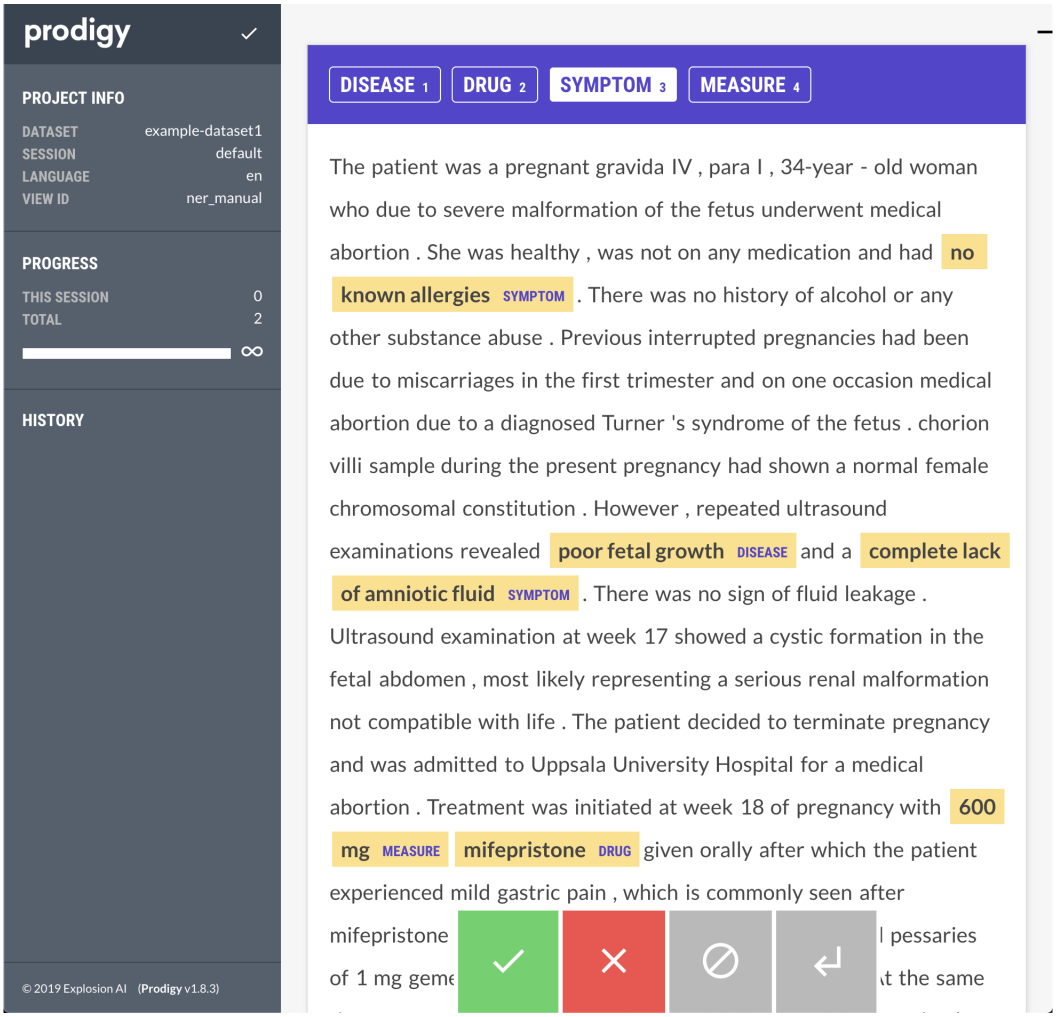

Figure 4.11: Screen-shot of prodigy.

4.12 tagtog

- Institution: tagtog
- URL: `http://www.tagtog.net/`
- Publication: [26]

Publication	
P1	The publication is from 2014.
P2	There are 14 citations for this publication in Google Scholar
P3	3 citations: for the corpora LocText [36, 27], IDP4, nala and nala_discoveries [25]. However, all these corpora were developed by the authors of the tool.

Technical	
T1	Being a commercial product, no information could be found about the date of the last version of the tool.
T2	Being a commercial product, no source code is available.
T3	Yes, tool is available on-line (via log in) but there are some restrictions for the free version, such as a maximum of two annotation projects.
T4	No need to install it.
T5	Documentation is good and clear.
T6	License is unknown
T7	Freely available with limitations, e.g. number of projects (only one), users (only one), entity types (up to three), among others

Data	
D1	Schema (document-level and entity-level labels, as well as relationships) can be defined in the Web interface.
D2	The tool accepts various formats [2], such as plain text, XML, PDF and HTML.
D3	The same is valid for output (cf. criterion D2).

	Functional
F1	Overlapping annotations are allowed, but not over the same span. However, maybe it would be possible using the entity labels (which are not clear to us) instead of the entity types.
F2	Yes, document-level annotation is possible without the need of highlighting a span of text.
F3	Yes, relation between two entities can be annotated.
F4	Yes, it seems to possible to import or define terminologies or ontologies to support the annotation, as carried out by the FlyBase curators [26].
F5	No, it does not seem to be possible to import pre-annotations produced by another tool.
F6	Yes, integration with PubMed is available but some documents (e.g., 11591455, 16897723) could not be found or loaded/parsed.
F7	Yes, the tool does support full text, even though it did not work for us (not even with the examples PMCID).
F8	Yes, documents can be saved at any time.
F9	Yes, it is possible to highlight entities.
F10	No, the multi-user feature is not available in the free version of the tool.
F11	No, given that no multi-user feature is available (cf. criterion F10), neither IAA is available.
F12	No, data cannot be private by using the free version of the tool. The user must pay for the on-premises version (a Docker image).
F13	Yes, text in Chinese was correctly displayed and annotated.

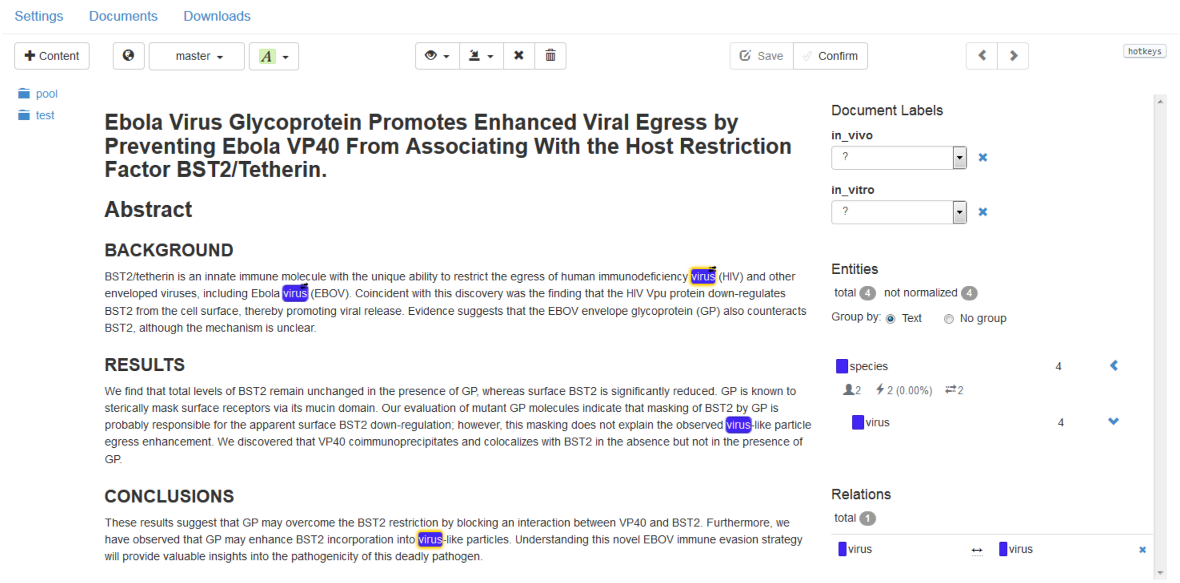

Figure 4.12: Screen-shot of tagtog.

4.13 TextAE

- Institution: Database Center for Life Science (DBCLS)
- URL: `http://textae.pubannotation.org/`
- Publication: -

Publication	
P1	No publication found
P2	We found 2 citations to the tool's URL.
P3	We found no publication describing a corpus development.

Technical	
T1	Last commit in GitHub was on March 15th, 2018.
T2	Yes, source code is available in GitHub.
T3	Yes, the tool is available on-line.
T4	No need to install it.
T5	Documentation is good and clear.
T6	MIT License
T7	Freely available in its all functionalities

Data	
D1	Schema (annotation labels) can be defined in the Web interface.
D2	JSON (PubAnnotation JSON Format) for document input.
D3	JSON (PubAnnotation JSON Format) for exporting annotations.

	Functional
F1	Overlapping annotations are allowed, but not over the same span.
F2	No annotations on document-level.
F3	Yes, it is possible to annotate relationships.
F4	No, it is not possible to import or define terminologies or ontologies to support the annotation.
F5	Yes, by importing documents and annotation as a JSON file.
F6	No integration to neither Medline nor PubMed, except with the use of PubAnnotation [3].
F7	No, long document can be uploaded but the JSON format does not allow break lines. Therefore, visualization of long documents is poor.
F8	Yes, partial annotation can be exported to a JSON file which can be later re-imported.
F9	Yes, it is possible to highlight entities.
F10	No, there is no login, thus, no support for user nor teams.
F11	No, no inter-annotation agreement available.
F12	Yes, data can be private if the system is installed locally.
F13	Text in Chinese were correctly displayed, however annotation only worked for the whole sequence of characters but not for a small subset of characters.

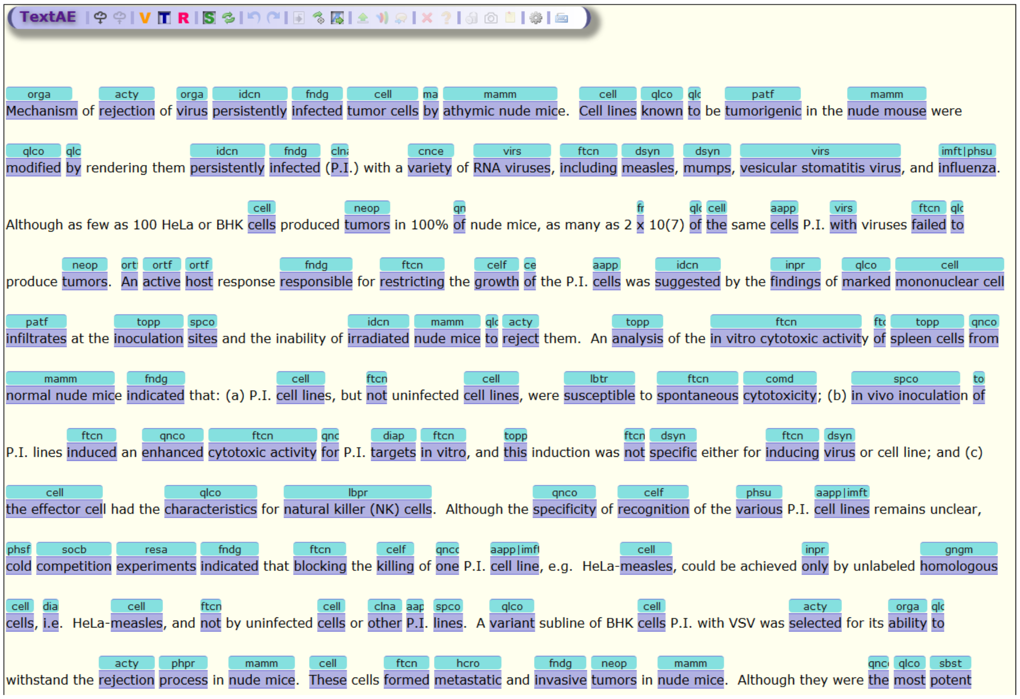

Figure 4.13: Screen-shot of TextAE.

4.14 WAT-SL

- Bauhaus-Universität Weimar (Germany)
- URL: `https://github.com/webis-de/wat`
- Publication: [49]

	Publication
P1	The publication is from 2016.
P2	We found four citations to the publication.
P3	We found one citation describing a corpus.

	Technical
T1	Last commit in GitHub was on July 9th, 2018.
T2	Yes, source code is available in GitHub.
T3	No, just a demo version is available on-line.
T4	It was easy to install it, it is only necessary to run a Java .jar file.
T5	Documentation is poor and limited to some .conf files.
T6	MIT License
T7	Freely available in its all functionalities

	Data
D1	Schema (annotation labels) is defined in a plain text .conf file.
D2	Documents are imported in plain text files.
D3	Annotations are exported in plain text (TAB-separated) files.

	Functional
F1	Overlapping annotations are not allowed, it is only possible to assign one label per segment.
F2	No, annotations on document-level are not possible.
F3	No, it is not possible to annotate relationships.
F4	No, the tool is not suitable for working with terminologies or ontologies to support the annotation.
F5	Yes, by importing the annotation in the plain text format.
F6	No integration to neither Medline nor PubMed.
F7	Yes, long documents can be imported and break lines are considered by the tool.
F8	Yes, partial annotations are supported.
F9	No, it is not possible to highlight arbitrary text spans.
F10	Yes, it is possible to define users and assign projects, but teams are not supported.
F11	Yes, inter-annotation agreement seems to be possible in the curator interface.
F12	Yes, data can be private because the tool needs to be installed locally.
F13	No, text in Chinese was not correctly displayed in the tool.

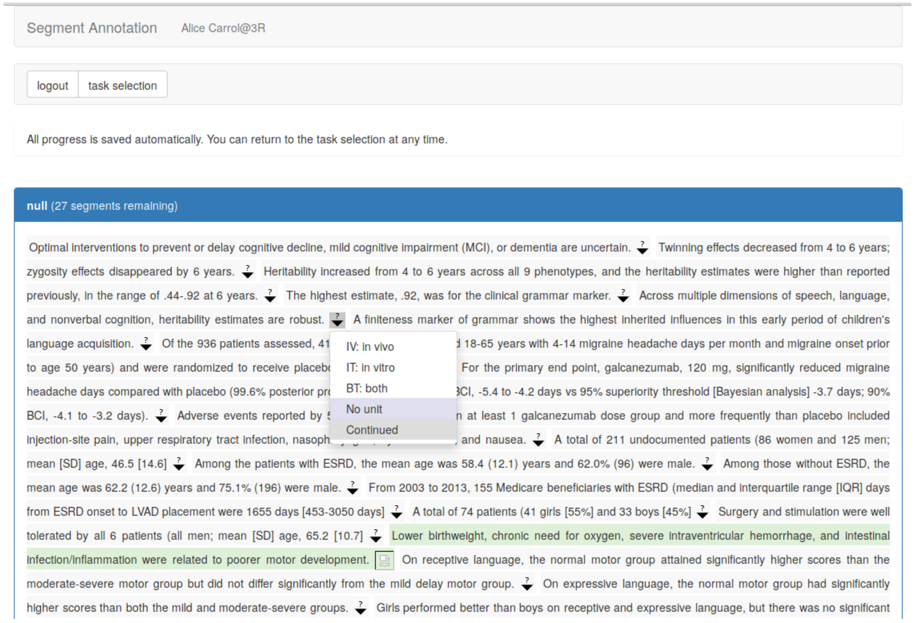

Figure 4.14: Screen-shot of WAT-SL.

4.15 WebAnno

- Institution: Technische Universität Darmstadt, Heidelberg University
- URL: `http://webanno.github.io`
- Publications: [32, 136]

Publication	
P1	Last publication from 2016, but three previous publications in 2014 and 2013.
P2	The four publications have a total of 126 citations (varying from only 9 citations, for the more recent one, to 76 citations for the oldest one)
P3	We found at least 13 citations for corpus development, namely, [103], [135], [143], [40], [137], [105], [29], [131], [73], [102], [140], [87], [110]

Technical	
T1	Last release (3.4.2) from June/2018.
T2	Yes, source code is available.
T3	No, the tool is not available on-line.
T4	Installing the tool requires some configuration (e.g., database) and dependencies.
T5	Documentation is very good and clear.
T6	Apache License v2.0
T7	Freely available in its all functionalities

Data	
D1	Schema can be configured in the GUI or JSON or TSV formats.
D2	Document can be imported in plain text, CoNLL, XML, TSV, among others.
D3	For export of annotations, it supports the same formats as for documents (cf. criterion D2).

Functional	
F1	Yes, multi-label annotations are possible.
F2	No, document-level annotation is not possible, unless if using a zero-width annotation.
F3	Yes, it is possible to create relations between annotations.
F4	Yes, terminologies can be imported as tag sets in the supported formats (cf. criterion D1).
F5	Yes, pre-annotation can be imported together with the documents in the supported formats (cf. criterion D2).
F6	No, there is no specific integration with neither Medline nor PubMed.
F7	Yes, full texts can be imported.
F8	Yes, it is possible to save documents partially.
F9	Yes, it is possible to highlight entities.
F10	There is support for users but not for teams.
F11	Yes, there is support for inter-annotator agreement.
F12	Yes, data can stay private as local installation of the tool is need.
F13	Yes, text in Chinese was correctly displayed.

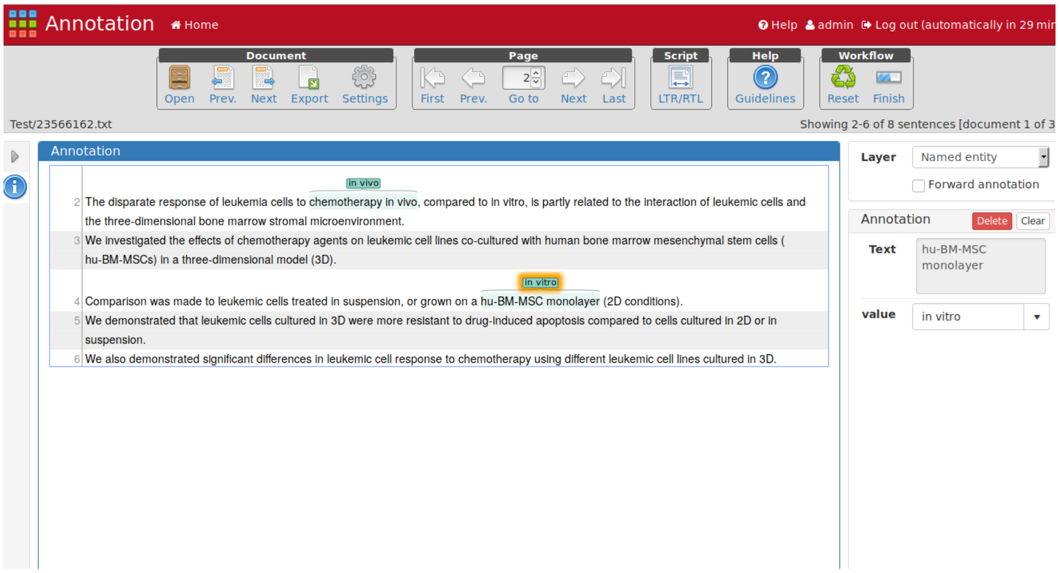

Figure 4.15: Screen-shot of WebAnno.

5 Details of the scores

5.1 Publication criteria

Tools	P1	P2	P3	Total Score
BioQRator	1.0	0.5	0.0	1.5
brat	1.0	1.0	1.0	3.0
Catma	0.0	0.0	0.0	0.0
Djangology	1.0	0.5	0.0	1.5
ezTag	1.0	0.0	0.0	1.0
FLAT	0.0	0.0	0.0	0.0
LightTag	0.0	0.0	0.0	0.0
MAT	0.0	0.0	0.0	0.0
MyMiner	1.0	0.5	0.0	1.5
PDFAnno	1.0	0.0	0.0	1.0
prodigy	0.0	0.0	0.0	0.0
tagtog	1.0	0.5	0.0	1.5
TextAE	0.0	0.0	0.0	0.0
WAT-SL	1.0	0.0	0.0	1.0
WebAnno	1.0	1.0	1.0	3.0

5.2 Technical criteria

Tools	T1	T2	T3	T4	T5	T6	T7	Total Score
BioQRator	0.5	0.0	1.0	1.0	1.0	0.0	1.0	4.5
brat	0.5	1.0	0.0	0.5	1.0	1.0	1.0	5.0
Catma	1.0	1.0	1.0	1.0	1.0	1.0	1.0	7.0
Djangology	0.5	0.5	0.0	0.5	0.5	0.0	1.0	3.0
ezTag	1.0	1.0	1.0	1.0	1.0	0.0	1.0	6.0
FLAT	1.0	1.0	1.0	1.0	1.0	1.0	1.0	7.0
LightTag	0.0	0.0	1.0	1.0	1.0	0.0	0.5	3.5
MAT	0.5	0.0	0.0	1.0	1.0	1.0	1.0	4.5
MyMiner	0.0	0.0	1.0	1.0	1.0	0.0	1.0	4.0
PDFAnno	1.0	1.0	1.0	1.0	0.5	1.0	1.0	6.5
prodigy	1.0	0.0	0.0	1.0	1.0	0.0	0.0	3.0
tagtog	0.0	0.0	0.5	1.0	1.0	0.0	0.5	3.0
TextAE	1.0	1.0	1.0	1.0	1.0	1.0	1.0	7.0
WAT-SL	1.0	1.0	0.0	1.0	0.5	1.0	1.0	5.5
WebAnno	1.0	1.0	0.0	0.0	1.0	1.0	1.0	5.0

5.3 Data criteria

Tools	D1	D2	D3	Total Score
BioQRator	1.0	1.0	1.0	3.0
brat	0.5	1.0	0.5	2.0
Catma	1.0	0.5	1.0	2.5
Djangology	1.0	0.5	0.5	2.0
ezTag	1.0	1.0	1.0	3.0
FLAT	1.0	1.0	1.0	3.0
LightTag	1.0	1.0	1.0	3.0
MAT	1.0	1.0	1.0	3.0
MyMiner	1.0	0.5	0.5	2.0
PDFAnno	1.0	1.0	1.0	3.0
prodigy	1.0	1.0	1.0	3.0
tagtog	1.0	1.0	1.0	3.0
TextAE	1.0	1.0	1.0	3.0
WAT-SL	0.5	0.5	0.5	1.5
WebAnno	1.0	1.0	1.0	3.0

5.4 Functional criteria

Tools	F1	F2	F3	F4	F5	F6	F7	F8	F9	F10	F11	F12	F13	Total Score
BioQRator	0.0	0.0	1.0	1.0	0.5	1.0	0.0	1.0	1.0	0.5	0.0	0.0	0.0	6.0
brat	1.0	0.0	1.0	1.0	0.5	0.0	1.0	1.0	1.0	0.5	0.5	1.0	1.0	9.5
Catma	1.0	0.0	0.0	0.0	0.5	0.0	0.5	1.0	1.0	0.5	0.0	1.0	1.0	6.5
Djangology	1.0	0.0	0.0	0.0	0.5	0.0	1.0	1.0	1.0	1.0	1.0	1.0	0.0	7.5
ezTag	0.0	0.0	0.0	1.0	1.0	1.0	1.0	1.0	1.0	0.5	0.0	1.0	0.0	7.5
FLAT	1.0	0.0	0.0	1.0	0.5	0.0	1.0	1.0	1.0	1.0	0.0	1.0	1.0	8.5
LightTag	1.0	0.0	1.0	0.0	0.5	0.0	1.0	1.0	1.0	1.0	1.0	0.0	1.0	8.5
MAT	1.0	1.0	1.0	0.0	0.5	0.0	1.0	0.5	1.0	0.0	0.0	1.0	1.0	8.0
MyMiner	0.0	1.0	0.5	0.0	0.5	1.0	0.0	0.5	1.0	0.0	0.5	0.0	1.0	6.0
PDFAnno	0.0	0.0	1.0	0.0	0.0	0.0	1.0	0.5	1.0	0.5	0.5	1.0	1.0	6.5
prodigy	0.0	1.0	0.0	1.0	1.0	0.0	1.0	1.0	1.0	0.5	0.0	1.0	1.0	8.5
tagtog	0.0	1.0	1.0	1.0	0.0	1.0	1.0	1.0	1.0	0.0	0.0	0.0	1.0	8.0
TextAE	0.5	0.0	1.0	0.0	1.0	0.5	0.0	0.5	1.0	0.0	0.0	1.0	0.5	6.0
WAT-SL	0.0	0.0	0.0	0.0	0.5	0.0	1.0	1.0	0.0	0.5	0.5	1.0	0.0	4.5
WebAnno	1.0	0.0	1.0	1.0	0.5	0.0	1.0	1.0	1.0	0.5	1.0	1.0	1.0	10.0

5.5 Ablation study

We investigated the changes in the score and in the ranking of the annotation when excluding each of the group of criteria that considered. Table 1 shows the variation in the ranks of each tool during the ablation study.

Tools	Average	Max	Min
BioQRator	10	12	8
brat	2.4	3	2
Catma	7.4	13	5
Djangology	11.6	8	13
ezTag	4	4	4
FLAT	3.4	5	1
LightTag	10.4	14	6
MAT	8.6	10	8
MyMiner	12.8	14	11
PDFAnno	5.8	11	2
prodigy	11.6	15	7
tagtog	8.8	12	3
TextAE	8.2	14	6
WAT-SL	13.8	15	9
WebAnno	1.2	2	1

Table 1: Average, maximum, and minimum ranks obtained by the annotation tools in the ablation study.

We present the different rankings according to the ablation study in Table 2.

Rank	All groups	w/o P	w/o T	w/o D	w/o F
1	WebAnno	FLAT	WebAnno	WebAnno	WebAnno
2	brat	WebAnno	brat	brat	PDFAnno
3	FLAT	brat	tagtog	FLAT	brat
4	ezTag	ezTag	ezTag	ezTag	ezTag
5	PDFAnno	Catma	FLAT	PDFAnno	FLAT
6	Catma	PDFAnno	LightTag	Catma	TextAE
7	TextAE	TextAE	prodigy	TextAE	Catma
8	MAT	MAT	Djangology	MAT	BioQRator
9	tagtog	LightTag	MAT	tagtog	WAT-SL
10	BioQRator	prodigy	BioQRator	BioQRator	MAT
11	LightTag	tagtog	PDFAnno	Djangology	MyMiner
12	prodigy	BioQRator	MyMiner	LightTag	tagtog
13	Djangology	Djangology	Catma	MyMiner	Djangology
14	MyMiner	MyMiner	TextAE	prodigy	LightTag
15	WAT-SL	WAT-SL	WAT-SL	WAT-SL	prodigy

Table 2: Different rankings of the annotation tools according to the group of criteria that was removed: publication (P), technical (T), data (D), or functional (F).

We present details of the scores when removing each of the groups in the tables below.

Tools	T	D	F	Total	Scores
BioQRator	4.5	3.0	6.0	13.5	0.59
brat	5.0	2.0	9.5	16.5	0.72
Catma	7.0	2.5	6.5	16	0.69
Djangology	3.0	2.0	7.5	12.5	0.54
ezTag	6.0	3.0	7.5	16.5	0.72
FLAT	7.0	3.0	8.5	18,5	0.80
LightTag	3.5	3.0	8.5	15	0.65
MAT	4.5	3.0	8.0	15.5	0.67
MyMiner	4.0	2.0	6.0	12	0.52
PDFAnno	6.5	3.0	6.5	16	0.69
prodigy	3.0	3.0	8.5	14.5	0.63
tagtog	3.0	3.0	8.0	14	0.61
TextAE	7.0	3.0	6.0	16	0.69
WAT-SL	5.5	1.5	4.5	11.5	0.50
WebAnno	5.0	3.0	10.0	18	0.78

Table 3: Total of points and scores when removing the publication criteria.

Tools	P	D	F	Total	Scores
BioQRator	1.5	3.0	6.0	10.5	0.55
brat	3.0	2.0	9.5	14.5	0.76
Catma	0.0	2.5	6.5	9	0.47
Djangology	1.5	2.0	7.5	11	0.58
ezTag	1.0	3.0	7.5	11.5	0.60
FLAT	0.0	3.0	8.5	11.5	0.60
LightTag	0.0	3.0	8.5	11.5	0.60
MAT	0.0	3.0	8.0	11	0.58
MyMiner	1.5	2.0	6.0	9.5	0.5
PDFAnno	1.0	3.0	6.5	10.5	0.55
prodigy	0.0	3.0	8.5	11.5	0.60
tagtog	1.5	3.0	8.0	12.5	0.66
TextAE	0.0	3.0	6.0	9	0.47
WAT-SL	1.0	1.5	4.5	7	0.37
WebAnno	3.0	3.0	10.0	16	0.84

Table 4: Total of points and scores when removing the technical criteria.

Tools	P	T	F	Total	Scores
BioQRator	1.5	4.5	6.0	12	0.52
brat	3.0	5.0	9.5	17.5	0.76
Catma	0.0	7.0	6.5	13.5	0.59
Djangology	1.5	3.0	7.5	12	0.52
ezTag	1.0	6.0	7.5	14.5	0.63
FLAT	0.0	7.0	8.5	15.5	0.67
LightTag	0.0	3.5	8.5	12	0.52
MAT	0.0	4.5	8.0	12.5	0.54
MyMiner	1.5	4.0	6.0	11.5	0.50
PDFAnno	1.0	6.5	6.5	14	0.61
prodigy	0.0	3.0	8.5	11.5	0.50
tagtog	1.5	3.0	8.0	12.5	0.54
TextAE	0.0	7.0	6.0	13	0.56
WAT-SL	1.0	5.5	4.5	11	0.48
WebAnno	3.0	5.0	10.0	18	0.78

Table 5: Total of points and scores when removing the data criteria.

Tools	P	T	D	Total	Scores
BioQRator	1.5	4.5	3.0	9	0.69
brat	3.0	5.0	2.0	10	0.77
Catma	0.0	7.0	2.5	9.5	0.73
Djangology	1.5	3.0	2.0	6.5	0.50
ezTag	1.0	6.0	3.0	10	0.77
FLAT	0.0	7.0	3.0	10	0.77
LightTag	0.0	3.5	3.0	6.5	0.50
MAT	0.0	4.5	3.0	7.5	0.58
MyMiner	1.5	4.0	2.0	7.5	0.58
PDFAnno	1.0	6.5	3.0	10.5	0.81
prodigy	0.0	3.0	3.0	6	0.46
tagtog	1.5	3.0	3.0	7.5	0.58
TextAE	0.0	7.0	3.0	10	0.77
WAT-SL	1.0	5.5	1.5	8	0.61
WebAnno	3.0	5.0	3.0	11	0.85

Table 6: Total of points and scores when removing the functional criteria.

References

[1] Abdulrahman K. AAlAbdulsalam, Jennifer H. Garvin, Andrew Redd, Marjorie E. Carter, Carol Sweeny, and Stephane M. Meystre. Automated extraction and classification of cancer stage mentions fromunstructured text fields in a central cancer registry. *AMIA Joint Summits on Translational Science proceedings. AMIA Joint Summits on Translational Science*, 2017:16–25, May 2018. 29888032[pmid].

[2] Saber A. Akhondi, Alexander G. Klenner, Christian Tyrchan, Anil K. Manchala, Kiran Boppana, Daniel Lowe, Marc Zimmermann, Sarma A. R. P. Jagarlapudi, Roger Sayle, Jan A. Kors, and Sorel Muresan. Annotated chemical patent corpus: A gold standard for text mining. *PLOS ONE*, 9(9):1–8, 09 2014.

[3] Saber A Akhondi, Hinnerk Rey, Markus Schwörer, Michael Maier, John Toomey, Heike Nau, Gabriele Ilchmann, Mark Sheehan, Matthias Irmer, Claudia Bobach, Marius Doornenbal, Michelle Gregory, and Jan A Kors. Automatic identification of relevant chemical compounds from patents. *Database*, 2019, 01 2019.

[4] Nestor Alvaro, Yusuke Miyao, and Nigel Collier. Twimed: Twitter and pubmed comparable corpus of drugs, diseases, symptoms, and their relations. *JMIR Public Health Surveill*, 3(2):e24, May 2017.

[5] Emilia Apostolova, Sean Neilan, Gary An, Noriko Tomuro, and Steven Lytinen. Djangology: A light-weight web-based tool for distributed collaborative text annotation. In Nicoletta Calzolari (Conference Chair), Khalid Choukri, Bente Maegaard, Joseph Mariani, Jan Odijk, Stelios Piperidis, Mike Rosner, and Daniel Tapias, editors, *Proceedings of the Seventh International Conference on Language Resources and Evaluation (LREC'10)*, Valletta, Malta, may 2010. European Language Resources Association (ELRA).

[6] Isabelle Augenstein, Mrinal Das, Sebastian Riedel, Lakshmi Vikraman, and Andrew McCallum. SemEval 2017 task 10: ScienceIE - extracting keyphrases and relations from scientific publications. In *Proceedings of the 11th International Workshop on Semantic Evaluation (SemEval-2017)*, pages 546–555, Vancouver, Canada, August 2017. Association for Computational Linguistics.

[7] Isabelle Augenstein, Mrinal Das, Sebastian Riedel, Lakshmi Vikraman, and Andrew McCallum. Semeval 2017 task 10: Scienceie - extracting keyphrases and relations from scientific publications. In *Proceedings of the 11th International Workshop on Semantic Evaluation (SemEval-2017)*, pages 546–555, Vancouver, Canada, August 2017. Association for Computational Linguistics.

[8] Abderrahim Ait Azzi, Houda Bouamor, and Sira Ferradans. The FinSBD-2019 shared task: Sentence boundary detection in PDF noisy text in the financial domain. In *Proceedings of the First Workshop on Financial Technology and Natural Language Processing*, pages 74–80, Macao, China, 12 August 2019.

[9] Michael Bada, Nicole Vasilevsky, Jr Baumgartner, William A, Melissa Haendel, and Lawrence E Hunter. Gold-standard ontology-based anatomical annotation in the CRAFT Corpus. *Database*, 2017, 12 2017.

[10] Michael Bada, Nicole Vasilevsky, Melissa Haendel, and Lawrence Hunter. Gold-standard ontology-based annotation of concepts in biomedical text in the craft corpus: Updates and extensions. In *Proceedings of the Joint International Conference on Biological Ontology and BioCreative*, Corvallis, Oregon, United States, 2016.

[11] Simon Baker, Imran Ali, Ilona Silins, Sampo Pyysalo, Yufan Guo, Johan Högberg, Ulla Stenius, and Anna Korhonen. Cancer Hallmarks Analytics Tool (CHAT): a text mining approach to organize and evaluate scientific literature on cancer. *Bioinformatics*, 33(24):3973–3981, 07 2017.

[12] Simon Baker, Douwe Kiela, and Anna Korhonen. Robust text classification for sparsely labelled data using multi-level embeddings. In *Proceedings of COLING 2016, the 26th International Conference on Computational Linguistics: Technical Papers*, pages 2333–2343, Osaka, Japan, December 2016. The COLING 2016 Organizing Committee.

[13] John A Bateman, Francisco OD Veloso, and Yan Ling Lau. On the track of visual style: a diachronic study of page composition in comics and its functional motivation. *Visual Communication*, 0(0):1470357219839101, 0.

[14] Michael Beißwenger, Thierry Chanier, Tomaž Erjavec, Darja Fišer, Axel Herold, Nikola Ljubešić, Harald Lüngen, Celine Poudat, Egon W. Stemle, Angelika Storrer, and Ciara Wigham. Closing a gap in the language resources landscape: Groundwork and best practices from projects on computer-mediated communication in four european countries. Selected papers from the CLARIN Annual Conference 2016, Aix-en-Provence, 26–28 October 2016, CLARIN Common Language Resources and Technology Infrastructure, pages 1 – 18, Linköping, 2017. Linköping University.

[15] Darina Benikova, Margot Mieskes, Christian M. Meyer, and Iryna Gurevych. Bridging the gap between extractive and abstractive summaries: Creation and evaluation of coherent extracts from heterogeneous sources. In *Proceedings of COLING 2016, the 26th International Conference on Computational Linguistics: Technical Papers*, pages 1039–1050, Osaka, Japan, December 2016. The COLING 2016 Organizing Committee.

[16] Steven Bethard and Jonathan Parker. A semantically compositional annotation scheme for time normalization. In *Proceedings of the Tenth International Conference on Language Resources and Evaluation (LREC 2016)*, pages 3779–3786, Portorož, Slovenia, May 2016. European Language Resources Association (ELRA).

[17] Thomas Bögel, Jannik Strötgen, Christoph Mayer, and Michael Gertz. A flexible nlp pipeline for computational narratology. In *1. Jahrestagung der Digital Humanities im deutschsprachigen Raum (DHd 2014), Passau, Germany*, 2014.

[18] K. Bontcheva and L. Derczynski. Chapter 6 - extracting information from social media with gate. In Emma L. Tonkin and Gregory J.L. Tourte, editors, *Working with Text*, Chandos Information Professional Series, pages 133 – 158. Chandos Publishing, 2016.

[19] Benjamin Boucher, Anna Y. Lee, Michael Hallett, and Sarah Jenna. Structural and functional characterization of a caenorhabditis elegans genetic interaction network within pathways. *PLOS Computational Biology*, 12(2):1–31, 02 2016.

[20] Melania Cabezas-García and Antonio San Martín. Semantic annotation to characterize contextual variation in terminological noun compounds: a pilot study. In *Proceedings of the 13th Workshop on Multiword Expressions (MWE 2017)*, pages 108–113, Valencia, Spain, April 2017. Association for Computational Linguistics.

[21] Arlene Casey, Bonnie Webber, and Dorota Glowacka. A framework for annotating 'related works' to support feedback to novice writers. In *Proceedings of the 13th Linguistic Annotation Workshop*, pages 90–99, Florence, Italy, August 2019. Association for Computational Linguistics.

[22] Sergio M. Castro, Eugene Tseytlin, Olga Medvedeva, Kevin Mitchell, Shyam Visweswaran, Tanja Bekhuis, and Rebecca S. Jacobson. Automated annotation and classification of bi-rads assessment from radiology reports. *Journal of Biomedical Informatics*, 69:177 – 187, 2017.

[23] Klara Ceberio, Itziar Aduriz, Arantza Díaz de Ilarraza, and Ines Garcia-Azkoaga. Coreferential relations in basque: The annotation process. *Journal of Psycholinguistic Research*, 47(2):325–342, Apr 2018.

[24] Juan Miguel Cejuela, Aleksandar Bojchevski, Carsten Uhlig, Rustem Bekmukhametov, Sanjeev Kumar Karn, Shpend Mahmuti, Ashish Baghudana, Ankit Dubey, Venkata P Satagopam, and Burkhard Rost. nala: text mining natural language mutation mentions. *Bioinformatics*, 33(12):1852–1858, 02 2017.

[25] Juan Miguel Cejuela, Aleksandar Bojchevski, Carsten Uhlig, Rustem Bekmukhametov, Sanjeev Kumar Karn, Shpend Mahmuti, Ashish Baghudana, Ankit Dubey, Venkata P. Satagopam, and Burkhard Rost. nala: text

mining natural language mutation mentions. *Bioinformatics*, 33(12):1852–1858, 2017.

[26] Juan Miguel Cejuela, Peter McQuilton, Laura Ponting, Steven J. Marygold, Raymund Stefancsik, Gillian H. Millburn, and Burkhard Rost. tagtog: interactive and text-mining-assisted annotation of gene mentions in plos full-text articles. *Database*, 2014:bau033, 2014.

[27] Juan Miguel Cejuela, Shrikant Vinchurkar, Tatyana Goldberg, Madhukar Sollepura Prabhu Shankar, Ashish Baghudana, Aleksandar Bojchevski, Carsten Uhlig, André Ofner, Pandu Raharja-Liu, Lars Juhl Jensen, and Burkhard Rost. LocText: relation extraction of protein localizations to assist database curation. *BMC Bioinformatics*, 19(1):15, Jan 2018.

[28] Lingzhen Chen, Alessandro Moschitti, Giuseppe Castellucci, Andrea Favalli, and Raniero Romagnoli. Transfer learning for industrial applications of named entity recognition. In *Proceedings of the 2nd Workshop on Natural Language for Artificial Intelligence (NL4AI 2018)*, pages 129–140, Trento, Italy, 2018.

[29] Çağrı Çöltekin, Ben Campbell, Erhard Hinrichs, and Heike Telljohann. Converting the TüBa-D/Z treebank of German to universal dependencies. In *Proceedings of the NoDaLiDa 2017 Workshop on Universal Dependencies (UDW 2017)*, pages 27–37. Association for Computational Linguistics, 2017.

[30] Joke Daems, Sonia Vandepitte, Robert J. Hartsuiker, and Lieve Macken. Identifying the machine translation error types with the greatest impact on post-editing effort. *Frontiers in Psychology*, 8:1282, 2017.

[31] Son Doan, Cleo K. Maehara, Juan D. Chaparro, Sisi Lu, Ruiling Liu, Amanda Graham, Erika Berry, Chun-Nan Hsu, John T. Kanegaye, David D. Lloyd, Lucila Ohno-Machado, Jane C. Burns, Adriana H. Tremoulet, and the Pediatric Emergency Medicine Kawasaki Disease Research Group. Building a natural language processing tool to identify patients with high clinical suspicion for kawasaki disease from emergency department notes. *Academic Emergency Medicine*, 23(5):628–636, 2016.

[32] Richard Eckart de Castilho, Éva Mújdricza-Maydt, Seid Muhie Yimam, Silvana Hartmann, Iryna Gurevych, Anette Frank, and Chris Biemann. A web-based tool for the integrated annotation of semantic and syntactic structures. In *Proceedings of the Workshop on Language Technology Resources and Tools for Digital Humanities (LT4DH)*, pages 76–84. The COLING 2016 Organizing Committee, 2016.

[33] Darja Fišer, Nikola Ljubešić, and Tomaž Erjavec. The janes project: language resources and tools for slovene user generated content. *Language Resources and Evaluation*, Sep 2018.

[34] Jennifer Hornung Garvin, Youngjun Kim, Glenn Temple Gobbel, Michael E Matheny, Andrew Redd, Bruce E Bray, Paul Heidenreich, Dan Bolton, Julia Heavirland, Natalie Kelly, Ruth Reeves, Megha Kalsy, Mary Kane Goldstein, and Stephane M Meystre. Automating quality measures for heart failure using natural language processing: A descriptive study in the department of veterans affairs. *JMIR Med Inform*, 6(1):e5, Jan 2018.

[35] Abbas Ghaddar and Phillippe Langlais. WikiCoref: An English coreference-annotated corpus of Wikipedia articles. In *Proceedings of the Tenth International Conference on Language Resources and Evaluation (LREC 2016)*, pages 136–142, Portorož, Slovenia, May 2016. European Language Resources Association (ELRA).

[36] Tatyana Goldberg, Shrikant Vinchurkar, Juan Miguel Cejuela, Lars Juhl Jensen, and Burkhard Rost. Linked annotations: a middle ground for manual curation of biomedical databases and text corpora. *BMC Proceedings*, 9(5):A4, Aug 2015.

[37] Cyril Grouin and Aurélie Névéol. De-identification of clinical notes in french: towards a protocol for reference corpus development. *Journal of Biomedical Informatics*, 50:151 – 161, 2014. Special Issue on Informatics Methods in Medical Privacy.

[38] Chulaka Gunasekara, Jonathan K. Kummerfeld, Lazaros Polymenakos, and Walter Lasecki. DSTC7 task 1: Noetic end-to-end response selection. In *Proceedings of the First Workshop on NLP for Conversational AI*, pages 60–67, Florence, Italy, August 2019. Association for Computational Linguistics.

[39] Erum Haris, Keng Hoon Gan, and Tien-Ping Tan. Spatial information extraction from travel narratives: Analysing the notion of co-occurrence indicating closeness of tourist places. *Journal of Information Science*, 0(0):0165551519837188, 0.

[40] Silvana Hartmann, Ilia Kuznetsov, Teresa Martin, and Iryna Gurevych. Out-of-domain framenet semantic role labeling. In *Proceedings of the 15th Conference of the European Chapter of the Association for Computational Linguistics (EACL 2017)*, pages 471–482. Association for Computational Linguistics, April 2017.

[41] Anna Hätty, Simon Tannert, and Ulrich Heid. Creating a gold standard corpus for terminological annotation from online forum data. In *Proceedings of Language, Ontology, Terminology and Knowledge Structures Workshop (LOTKS 2017)*, Montpellier, France, September 2017. Association for Computational Linguistics.

[42] Aron Henriksson, Maria Kvist, Hercules Dalianis, and Martin Duneld. Identifying adverse drug event information in clinical notes with distributional semantic representations of context. *Journal of Biomedical Informatics*, 57:333 – 349, 2015.

[43] Na Hong, Dingcheng Li, Yue Yu, Qiongying Xiu, Hongfang Liu, and Guoqian Jiang. A computational framework for converting textual clinical diagnostic criteria into the quality data model. *Journal of Biomedical Informatics*, 63:11 – 21, 2016.

[44] P. Jorge, Martín Pérez-Pérez, Gael Pérez Rodríguez, Florentino Fdez-Riverola, Maria Olívia Pereira, and Anália Lourenço. Construction of antimicrobial peptide-drug combination networks from scientific literature based on a semi-automated curation workflow. 2016.

[45] Meizhi Ju, Andrea D Short, Paul Thompson, Nawar Diar Bakerly, Georgios V Gkoutos, Loukia Tsaprouni, and Sophia Ananiadou. Annotating and detecting phenotypic information for chronic obstructive pulmonary disease. *JAMIA Open*, 2(2):261–271, 04 2019.

[46] David Jurgens, Srijan Kumar, Raine Hoover, Dan McFarland, and Dan Jurafsky. Measuring the evolution of a scientific field through citation frames. *Transactions of the Association for Computational Linguistics*, 6:391–406, 2018.

[47] Tian Kang, Shaodian Zhang, Youlan Tang, Gregory W Hruby, Alexander Rusanov, Noémie Elhadad, and Chunhua Weng. EliIE: An open-source information extraction system for clinical trial eligibility criteria. *Journal of the American Medical Informatics Association*, 24(6):1062–1071, 04 2017.

[48] Sarvnaz Karimi, Alejandro Metke-Jimenez, Madonna Kemp, and Chen Wang. Cadec: A corpus of adverse drug event annotations. *Journal of Biomedical Informatics*, 55:73 – 81, 2015.

[49] Johannes Kiesel, Henning Wachsmuth, Khalid Al Khatib, and Benno Stein. WAT-SL: A customizable web annotation tool for segment labeling. In *Proceedings of the 15th Conference of the European Chapter of the Association for Computational Linguistics, EACL 2017, Valencia, Spain, April 3-7, 2017, Software Demonstrations*, pages 13–16, 2017.

[50] Halil Kilicoglu, Asma Ben Abacha, Yassine Mrabet, Sonya E. Shooshan, Laritza Rodriguez, Kate Masterton, and Dina Demner-Fushman. Semantic annotation of consumer health questions. *BMC Bioinformatics*, 19(1):34, 2018.

[51] Halil Kilicoglu, Graciela Rosemblat, Marcelo Fiszman, and Thomas C. Rindflesch. Sortal anaphora resolution to enhance relation extraction from biomedical literature. *BMC Bioinformatics*, 17(1):163, 2016.

[52] Halil Kilicoglu, Graciela Rosemblat, and Thomas C. Rindflesch. Assigning factuality values to semantic relations extracted from biomedical research literature. *PLOS ONE*, 12(7):1–20, 07 2017.

[53] Evgeny Kim and Roman Klinger. Who feels what and why? annotation of a literature corpus with semantic roles of emotions. In *Proceedings of the 27th International Conference on Computational Linguistics*, pages 1345–1359, Santa Fe, New Mexico, USA, August 2018. Association for Computational Linguistics.

[54] Evgeny Kim and Roman Klinger. Frowning Frodo, wincing Leia, and a seriously great friendship: Learning to classify emotional relationships of fictional characters. In *Proceedings of the 2019 Conference of the North American Chapter of the Association for Computational Linguistics: Human Language Technologies, Volume 1 (Long and Short Papers)*, pages 647–653, Minneapolis, Minnesota, June 2019. Association for Computational Linguistics.

[55] Sunghwan Mac Kim and Steve Cassidy. Finding names in trove: Named entity recognition for australian historical newspapers. In *Proceedings of the Australasian Language Technology Association Workshop 2015*, pages 57–65, Parramatta, Australia, December 2015.

[56] Anna Koroleva and Patrick Paroubek. Annotating spin in biomedical scientific publications : the case of random controlled trials (RCTs). In *Proceedings of the Eleventh International Conference on Language Resources and Evaluation (LREC-2018)*, Miyazaki, Japan, May 2018. European Languages Resources Association (ELRA).

[57] Martin Krallinger, Florian Leitner, Obdulia Rabal, Miguel Vazquez, Julen Oyarzabal, and Alfonso Valencia. Overview of the chemical compound and drug name recognition (chemdner) task. In *In BioCreative Challenge Evaluation Workshop*, page 2, 2013.

[58] Martin Krallinger, Obdulia Rabal, Florian Leitner, Miguel Vazquez, David Salgado, Zhiyong Lu, Robert Leaman, Yanan Lu, Donghong Ji, Daniel M. Lowe, Roger A. Sayle, Riza Theresa Batista-Navarro, Rafal Rak, Torsten Huber, Tim Rocktäschel, Sérgio Matos, David Campos, Buzhou Tang, Hua Xu, Tsendsuren Munkhdalai, Keun Ho Ryu, S. V. Ramanan, Senthil Nathan, Slavko Zitnik, Marko Bajec, Lutz Weber, Matthias Irmer, Saber A. Akhondi, Jan A. Kors, Shuo Xu, Xin An, Utpal Kumar Sikdar, Asif Ekbal, Masaharu Yoshioka, Thaer M. Dieb, Miji Choi, Karin Verspoor, Madian Khabsa, C. Lee Giles, Hongfang Liu, Komandur Elayavilli Ravikumar, Andre Lamurias, Francisco M. Couto, Hong-Jie Dai, Richard Tzong-Han Tsai, Caglar Ata, Tolga Can, Anabel Usié, Rui Alves, Isabel Segura-Bedmar, Paloma Martínez, Julen Oyarzabal, and Alfonso Valencia. The chemdner corpus of chemicals and drugs and its annotation principles. *Journal of Cheminformatics*, 7(1):S2, 2015.

[59] Parul Kudtarkar and R. Andrew Cameron. Echinobase: an expanding resource for echinoderm genomic information. *Database*, 2017, 09 2017.

[60] Jonathan K. Kummerfeld, Sai R. Gouravajhala, Joseph J. Peper, Vignesh Athreya, Chulaka Gunasekara, Jatin Ganhotra, Siva Sankalp Patel, Lazaros C Polymenakos, and Walter Lasecki. A large-scale corpus for conversation disentanglement. In *Proceedings of the 57th Annual Meeting of the Association for Computational Linguistics*, pages 3846–3856, Florence, Italy, July 2019. Association for Computational Linguistics.

[61] Dongseop Kwon, Sun Kim, Soo-Yong Shin, Andrew Chatr-aryamontri, and W. John Wilbur. Assisting manual literature curation for protein-protein interactions using BioQRator. *Database*, 2014:bau067, 2014.

[62] Dongseop Kwon, Sun Kim, Chih-Hsuan Wei, Robert Leaman, and Zhiyong Lu. eztag: tagging biomedical concepts via interactive learning. *Nucleic Acids Research*, 46(W1):W523–W529, 2018.

[63] Ekaterina Lapshinova-Koltunski, Anna Nedoluzhko, and Kerstin Anna Kunz. Across languages and genres: Creating a universal annotation scheme for textual relations. In *Proceedings of The 9th Linguistic Annotation Workshop*, pages 168–177, Denver, Colorado, USA, June 2015. Association for Computational Linguistics.

[64] Kristin Larsson, Simon Baker, Ilona Silins, Yufan Guo, Ulla Stenius, Anna Korhonen, and Marika Berglund. Text mining for improved exposure assessment. *PLOS ONE*, 12(3):1–21, 03 2017.

[65] Florian Leitner, Martin Krallinger, Sushil Tripathi, Martin Kuiper, Astrid Laegreid, and Alfonso Valencia. Mining cis-regulatory transcription networks from literature. In *Proceedings BioLINK SIG*, pages 5–12, 2013.

[66] Jiao Li, Yueping Sun, Robin J. Johnson, Daniela Sciaky, Chih-Hsuan Wei, Robert Leaman, Allan Peter Davis, Carolyn J. Mattingly, Thomas C. Wiegers, and Zhiyong Lu. BioCreative V CDR task corpus: a resource for chemical disease relation extraction. *Database*, 2016, 05 2016.

[67] Qi Li, Eric S. Kirkendall, Eric S. Hall, Yizhao Ni, Todd Lingren, Megan Kaiser, Nataline Lingren, Haijun Zhai, Imre Solti, and Kristin Melton. Automated detection of medication administration errors in neonatal intensive care. *Journal of Biomedical Informatics*, 57:124 – 133, 2015.

[68] Qi Li, Stephen Andrew Spooner, Megan Kaiser, Nataline Lingren, Jessica Robbins, Todd Lingren, Huaxiu Tang, Imre Solti, and Yizhao Ni. An end-to-end hybrid algorithm for automated medication discrepancy detection. *BMC Medical Informatics and Decision Making*, 15(1):37, 2015.

[69] Yang Liu and Amir Zeldes. Discourse relations and signaling information: Anchoring discourse signals in rst-dt. In *Proceedings of the Society for*

Computation in Linguistics (SCiL) 2019, pages 314–317., New York, USA, 2019.

[70] Christina Lohr, Stephanie Luther, Franz Matthies, Luise Modersohn, Danny Ammon, Kutaiba Saleh, Andreas G. Henkel, Michael Kiehntopf, and Udo Hahn. Cda-compliant section annotation of german-language discharge summaries: Guideline development, annotation campaign, section classification. *AMIA ... Annual Symposium proceedings. AMIA Symposium*, 2018:770–779, Dec 2018. 30815119[pmid].

[71] Michał Marcińczuk, Marcin Oleksy, and Jan Kocoń. Inforex — a collaborative system for text corpora annotation and analysis. In *Proceedings of the International Conference Recent Advances in Natural Language Processing, RANLP 2017*, pages 473–482, Varna, Bulgaria, September 2017. INCOMA Ltd.

[72] Héctor Martínez Alonso, Anders Johannsen, Sussi Olsen, Sanni Nimb, Nicolai Hartvig Sørensen, Anna Braasch, Anders Søgaard, and Bolette Sandford Pedersen. Supersense tagging for Danish. In *Proceedings of the 20th Nordic Conference of Computational Linguistics (NODALIDA 2015)*, pages 21–29, Vilnius, Lithuania, May 2015. Linköping University Electronic Press, Sweden.

[73] Héctor Martínez Alonso, Anders Johannsen, Sussi Olsen, Sanni Nimb, Nicolai Hartvig Sørensen, Anna Braasch, Anders Søgaard, and Bolette Sandford Pedersen. Supersense tagging for danish. In *Proceedings of the 20th Nordic Conference of Computational Linguistics, NODALIDA 2015, May 11-13, 2015, Vilnius, Lithuania*, number 109, pages 21–29. Linköping University Electronic Press, Linköpings universitet, 2015.

[74] Pierre André Ménard, Sylvie Ratté, Geneviève Parent, and Franck Barbedor. Manual and automatic annotation of meeting reports with young offenders for quality assessment of interventions. In *Proceedings of the Workshop on Annotation in Digital Humanities*, Sofia, Bulgaria, 2018.

[75] Claudiu Mihăilă, Tomoko Ohta, Sampo Pyysalo, and Sophia Ananiadou. BioCause: Annotating and analysing causality in the biomedical domain. *BMC Bioinformatics*, 14(1):2, Jan 2013.

[76] Alba Milà-Garcia. Pragmatic annotation for a multi-layered analysis of speech acts: A methodological proposal. *Corpus Pragmatics*, 2(3):265–287, Sep 2018.

[77] Ashutosh Modi, Tatjana Anikina, Simon Ostermann, and Manfred Pinkal. Inscript: Narrative texts annotated with script information. *CoRR*, abs/1703.05260, 2017.

[78] H.-M. Müller, K. M. Van Auken, Y. Li, and P. W. Sternberg. Textpresso central: a customizable platform for searching, text mining, viewing, and curating biomedical literature. *BMC Bioinformatics*, 19(1):94, 2018.

[79] Sheshera Mysore, Zachary Jensen, Edward Kim, Kevin Huang, Haw-Shiuan Chang, Emma Strubell, Jeffrey Flanigan, Andrew McCallum, and Elsa Olivetti. The materials science procedural text corpus: Annotating materials synthesis procedures with shallow semantic structures. In *Proceedings of the 13th Linguistic Annotation Workshop*, pages 56–64, Florence, Italy, August 2019. Association for Computational Linguistics.

[80] Mardi J. Nenni, Malcolm E. Fisher, Christina James-Zorn, Troy J. Pells, Virgilio Ponferrada, Stanley Chu, Joshua D. Fortriede, Kevin A. Burns, Ying Wang, Vaneet S. Lotay, Dong Zhou Wang, Erik Segerdell, Praneet Chaturvedi, Kamran Karimi, Peter D. Vize, and Aaron M. Zorn. Xenbase: Facilitating the use of xenopus to model human disease. *Frontiers in Physiology*, 10:154, 2019.

[81] Joakim Nivre, Marie-Catherine de Marneffe, Filip Ginter, Yoav Goldberg, Jan Hajič, Christopher D. Manning, Ryan McDonald, Slav Petrov, Sampo Pyysalo, Natalia Silveira, Reut Tsarfaty, and Daniel Zeman. Universal dependencies v1: A multilingual treebank collection. In *Proceedings of the Tenth International Conference on Language Resources and Evaluation (LREC 2016)*, pages 1659–1666, Portorož, Slovenia, May 2016. European Language Resources Association (ELRA).

[82] Tomoko Ohta, Sampo Pyysalo, Jun'ichi Tsujii, and Sophia Ananiadou. Open-domain anatomical entity mention detection. In *Proceedings of the Workshop on Detecting Structure in Scholarly Discourse*, ACL '12, pages 27–36, Stroudsburg, PA, USA, 2012. Association for Computational Linguistics.

[83] Maite Oronoz, Koldo Gojenola, Alicia Pérez, Arantza Díaz de Ilarraza, and Arantza Casillas. On the creation of a clinical gold standard corpus in spanish: Mining adverse drug reactions. *Journal of Biomedical Informatics*, 56:318 – 332, 2015.

[84] Ruth Page. Group selfies and snapchat: From sociality to synthetic collectivisation. *Discourse, Context Media*, 28:79 – 92, 2019.

[85] Frédéric Papazian, Robert Bossy, and Claire Nédellec. AlvisAE: a collaborative web text annotation editor for knowledge acquisition. In *Proceedings of the Sixth Linguistic Annotation Workshop*, pages 149–152, Jeju, Republic of Korea, July 2012. Association for Computational Linguistics.

[86] Bolette Pedersen, Anna Braasch, Anders Johannsen, Héctor Martínez Alonso, Sanni Nimb, Sussi Olsen, Anders Søgaard, and Nicolai Hartvig Sørensen. The SemDaX corpus — sense annotations with scalable sense inventories. In *Proceedings of the Tenth International Conference on Language Resources and Evaluation (LREC 2016)*, pages 842–847, Portorož, Slovenia, May 2016. European Language Resources Association (ELRA).

[87] Bolette Pedersen, Anna Braasch, Anders Johannsen, Héctor Martínez Alonso, Sanni Nimb, Sussi Olsen, Anders Søgaard, and Nicolai Hartvig Sørensen. The SemDaX corpus — sense annotations with scalable sense inventories. In Nicoletta Calzolari (Conference Chair), Khalid Choukri, Thierry Declerck, Sara Goggi, Marko Grobelnik, Bente Maegaard, Joseph Mariani, Helene Mazo, Asuncion Moreno, Jan Odijk, and Stelios Piperidis, editors, *Proceedings of the Tenth International Conference on Language Resources and Evaluation (LREC 2016)*, Paris, France, may 2016. European Language Resources Association (ELRA).

[88] Crosthwaite Peter, Choy Lavigne L.Y., and Bae Yeonsuk. *opli*, volume 2, chapter 'Almost people': A Learner Corpus Account of L2 Use and Misuse of Non-numerical Quantification. 2019 2016. 1.

[89] Massimo Poesio, Yulia Grishina, Varada Kolhatkar, Nafise Moosavi, Ina Roesiger, Adam Roussel, Fabian Simonjetz, Alexandra Uma, Olga Uryupina, Juntao Yu, and Heike Zinsmeister. Anaphora resolution with the ARRAU corpus. In *Proceedings of the First Workshop on Computational Models of Reference, Anaphora and Coreference*, pages 11–22, New Orleans, Louisiana, June 2018. Association for Computational Linguistics.

[90] Maria Pontiki, Dimitris Galanis, Haris Papageorgiou, Ion Androutsopoulos, Suresh Manandhar, Mohammad AL-Smadi, Mahmoud Al-Ayyoub, Yanyan Zhao, Bing Qin, Orphée De Clercq, Véronique Hoste, Marianna Apidianaki, Xavier Tannier, Natalia Loukachevitch, Evgeniy Kotelnikov, Nuria Bel, Salud María Jiménez-Zafra, and Gülşen Eryiğit. SemEval-2016 task 5: Aspect based sentiment analysis. In *Proceedings of the 10th International Workshop on Semantic Evaluation (SemEval-2016)*, pages 19–30, San Diego, California, June 2016. Association for Computational Linguistics.

[91] Maria Pontiki, Dimitris Galanis, Haris Papageorgiou, Ion Androutsopoulos, Suresh Manandhar, Mohammed AL-Smadi, Mahmoud Al-Ayyoub, Yanyan Zhao, Bing Qin, Orphée De Clercq, Veronique Hoste, Marianna Apidianaki, Xavier Tannier, Natalia Loukachevitch, Evgeniy Kotelnikov, Núria Bel, Salud Maria Jiménez-Zafra, and Gülşen Eryiğit. Semeval-2016 task 5: aspect based sentiment analysis. In *Proceedings of the 10th International Workshop on Semantic Evaluation (SemEval-2016)*, pages 19–30. Association for Computational Linguistics, 2016.

[92] Sylvain Poux, Cecilia N Arighi, Michele Magrane, Alex Bateman, Chih-Hsuan Wei, Zhiyong Lu, Emmanuel Boutet, Hema Bye-A-Jee, Maria Livia Famiglietti, Bernd Roechert, and The UniProt Consortium. On expert curation and scalability: UniProtKB/Swiss-Prot as a case study. *Bioinformatics*, 33(21):3454–3460, 07 2017.

[93] James Pustejovsky, Parisa Kordjamshidi, Marie-Francine Moens, Aaron Levine, Seth Dworman, and Zachary Yocum. SemEval-2015 task 8:

SpaceEval. In *Proceedings of the 9th International Workshop on Semantic Evaluation (SemEval 2015)*, pages 884–894, Denver, Colorado, June 2015. Association for Computational Linguistics.

[94] Sampo Pyysalo, Tomoko Ohta, Makoto Miwa, Han-Cheol Cho, Jun'ichi Tsujii, and Sophia Ananiadou. Event extraction across multiple levels of biological organization. *Bioinformatics*, 28(18):i575–i581, 2012.

[95] Alicia Pérez, Rebecka Weegar, Arantza Casillas, Koldo Gojenola, Maite Oronoz, and Hercules Dalianis. Semi-supervised medical entity recognition: A study on spanish and swedish clinical corpora. *Journal of Biomedical Informatics*, 71:16 – 30, 2017.

[96] Majid Rastegar-Mojarad, Sijia Liu, Yanshan Wang, Naveed Afzal, Liwei Wang, Feichen Shen, Sunyang Fu, and Hongfang Liu. Biocreative/ohnlp challenge 2018. In *Proceedings of the 2018 ACM International Conference on Bioinformatics, Computational Biology, and Health Informatics*, BCB '18, pages 575–575, New York, NY, USA, 2018. ACM.

[97] Ayla Rigouts Terryn, Veronique Hoste, Joost Buysschaert, Robert Vander Stichele, Elise Van Campen, and Els Lefever. Validating multilingual hybrid automatic term extraction for search engine optimisation: the use case of ebm-guidelines. *Argentinian Journal of Applied Linguistics*, 7(1):93–108, 2019.

[98] Ryan Rivas, Niloofar Montazeri, Nhat XT Le, and Vagelis Hristidis. Automatic classification of online doctor reviews: Evaluation of text classifier algorithms. *J Med Internet Res*, 20(11):e11141, Nov 2018.

[99] Crosthwaite Peter Robert. *cllt*, volume 0, chapter Definite article bridging relations in L2: A learner corpus study. 2019 2016. 0.

[100] Julia Romberg. GDWDS: first insights from a student-based key phrase annotation process of medical information needs on a novel german diabetes web data set. In *Proceedings of the 30th GI-Workshop Grundlagen von Datenbanken, Wuppertal, Germany, May 22-25, 2018.*, pages 89–94, 2018.

[101] Rachel Rudinger, Vera Demberg, Ashutosh Modi, Benjamin Van Durme, and Manfred Pinkal. Learning to predict script events from domain-specific text. In *Proceedings of the Fourth Joint Conference on Lexical and Computational Semantics*, pages 205–210, Denver, Colorado, June 2015. Association for Computational Linguistics.

[102] Rachel Rudinger, Vera Demberg, Ashutosh Modi, Benjamin Van Durme, and Manfred Pinkal. Learning to predict script events from domain-specific text. In *Proceedings of the Fourth Joint Conference on Lexical and Computational Semantics*, pages 205–210, Denver, Colorado, June 2015. Association for Computational Linguistics.

[103] Björn Rudzewitz, Ramon Ziai, Kordula De Kuthy, and Detmar Meurers. Developing a web-based workbook for english supporting the interaction of students and teachers. In *Proceedings of the Joint 6th Workshop on NLP for Computer Assisted Language Learning and 2nd Workshop on NLP for Research on Language Acquisition at NoDaLiDa, Gothenburg, 22nd May 2017*, number 134, pages 36–46. Linköping University Electronic Press, Linköpings universitet, 2017.

[104] Jawad Sadek and Farid Meziane. Learning causality for arabic - proclitics. *Procedia Computer Science*, 142:141 – 149, 2018. Arabic Computational Linguistics.

[105] Driss Sadoun. A semi automatic annotation approach for ontological and terminological knowledge acquisition. In *Proceedings of the 5th International Workshop on Computational Terminology (Computerm2016)*, pages 110–120, Osaka, Japan, December 2016. The COLING 2016 Organizing Committee.

[106] David Salgado, Martin Krallinger, Marc Depaule, Elodie Drula, Ashish V. Tendulkar, Florian Leitner, Alfonso Valencia, and Christophe Marcelle. MyMiner: a web application for computer-assisted biocuration and text annotation. *Bioinformatics*, 28(17):2285–2287, 2012.

[107] Magali Sanches Duran and Sandra Aluísio. Automatic generation of a lexical resource to support semantic role labeling in Portuguese. In *Proceedings of the Fourth Joint Conference on Lexical and Computational Semantics*, pages 216–221, Denver, Colorado, June 2015. Association for Computational Linguistics.

[108] Aleksandar Savkov, John Carroll, Rob Koeling, and Jackie Cassell. Annotating patient clinical records with syntactic chunks and named entities: the harvey corpus. *Language Resources and Evaluation*, 50(3):523–548, Sep 2016.

[109] Tatjana Scheffler and Manfred Stede. Adding semantic relations to a large-coverage connective lexicon of German. In *Proceedings of the Tenth International Conference on Language Resources and Evaluation (LREC 2016)*, pages 1008–1013, Portorož, Slovenia, May 2016. European Language Resources Association (ELRA).

[110] Tatjana Scheffler and Manfred Stede. Adding semantic relations to a large-coverage connective lexicon of german. In Nicoletta Calzolari (Conference Chair), Khalid Choukri, Thierry Declerck, Sara Goggi, Marko Grobelnik, Bente Maegaard, Joseph Mariani, Helene Mazo, Asuncion Moreno, Jan Odijk, and Stelios Piperidis, editors, *Proceedings of the Tenth International Conference on Language Resources and Evaluation (LREC 2016)*, Paris, France, may 2016. European Language Resources Association (ELRA).

[111] Ayush Singhal, Michael Simmons, and Zhiyong Lu. Text mining for precision medicine: automating disease-mutation relationship extraction from biomedical literature. *Journal of the American Medical Informatics Association*, 23(4):766–772, 04 2016.

[112] Ander Soraluze, Olatz Arregi, Xabier Arregi, Arantza Díaz de Ilarraza, Mijail Kabadjov, and Massimo Poesio. Coreference resolution for the basque language with BART. In *Proceedings of the Workshop on Coreference Resolution Beyond OntoNotes (CORBON 2016)*, pages 67–73, San Diego, California, June 2016. Association for Computational Linguistics.

[113] Christian Stab and Iryna Gurevych. Parsing argumentation structures in persuasive essays. *Computational Linguistics*, 43(3):619–659, 2017.

[114] Sanja Štajner, Nicole Baerg, Simone Paolo Ponzetto, and Heiner Stuckenschmidt. Automatic detection of speculation in policy statements. In *NLP+CSS workshop at Web Science 2016 : May 22, 2016, Hannover, Germany*, pages 1–5, New York, NY, 2016. ACM. Nicht im WebSci '16 Proceedings-Band : http://dl.acm.org/citation.cfm?id=2908131 bzw.. http://www.aclweb.org/anthology/W16-5600.

[115] Pontus Stenetorp, Sampo Pyysalo, Goran Topić, Tomoko Ohta, Sophia Ananiadou, and Jun'ichi Tsujii. brat: A web-based tool for nlp-assisted text annotation. In *Proceedings of the Demonstrations at the 13th Conference of the European Chapter of the Association for Computational Linguistics*, EACL '12, pages 102–107, Stroudsburg, PA, USA, 2012. Association for Computational Linguistics.

[116] Amber Stubbs and Özlem Uzuner. Annotating longitudinal clinical narratives for de-identification: The 2014 i2b2/uthealth corpus. *Journal of Biomedical Informatics*, 58:S20 – S29, 2015. Proceedings of the 2014 i2b2/UTHealth Shared-Tasks and Workshop on Challenges in Natural Language Processing for Clinical Data.

[117] Amber Stubbs and Özlem Uzuner. Annotating risk factors for heart disease in clinical narratives for diabetic patients. *Journal of Biomedical Informatics*, 58:S78 – S91, 2015. Proceedings of the 2014 i2b2/UTHealth Shared-Tasks and Workshop on Challenges in Natural Language Processing for Clinical Data.

[118] Hanna Suominen, Liyuan Zhou, Lorraine Goeuriot, and Liadh Kelly. Task 1 of the clef ehealth evaluation lab 2016: Handover information extraction. In *Working Notes of CLEF 2016 - Conference and Labs of the Evaluation forum*, pages 1–14, Evora, Portugal, 2016.

[119] Dóra Szakonyi, Sofie Van Landeghem, Katja Baerenfaller, Lieven Baeyens, Jonas Blomme, Rubén Casanova-Sáez, Stefanie De Bodt, David Esteve-Bruna, Fabio Fiorani, Nathalie Gonzalez, Jesper Grønlund, Richard G.H. Immink, Sara Jover-Gil, Asuka Kuwabara, Tamara Muñoz-Nortes,

Aalt D.J. van Dijk, David Wilson-Sánchez, Vicky Buchanan-Wollaston, Gerco C. Angenent, Yves Van de Peer, Dirk Inzé, José Luis Micol, Wilhelm Gruissem, Sean Walsh, and Pierre Hilson. The knownleaf literature curation system captures knowledge about arabidopsis leaf growth and development and facilitates integrated data mining. *Current Plant Biology*, 2:1 – 11, 2015.

[120] Paul Thompson, Riza Theresa Batista-Navarro, Georgios Kontonatsios, Jacob Carter, Elizabeth Toon, John McNaught, Carsten Timmermann, Michael Worboys, and Sophia Ananiadou. Text mining the history of medicine. *PLOS ONE*, 11(1):1–33, 01 2016.

[121] Nhung T.H. Nguyen, Roselyn S. Gabud, and Sophia Ananiadou. Copious: A gold standard corpus of named entities towards extracting species occurrence from biodiversity literature. *Biodiversity Data Journal*, 7:e29626, 2019.

[122] Olga Uryupina, Ron Artstein, Antonella Bristot, Federica Cavicchio, Kepa Rodriguez, and Massimo Poesio. ARRAU: Linguistically-motivated annotation of anaphoric descriptions. In *Proceedings of the Tenth International Conference on Language Resources and Evaluation (LREC 2016)*, pages 2058–2062, Portorož, Slovenia, May 2016. European Language Resources Association (ELRA).

[123] Minoska Valli, Nadine E. Tatto, Armin Peymann, Clemens Gruber, Nils Landes, Heinz Ekker, Gerhard G. Thallinger, Diethard Mattanovich, Brigitte Gasser, and Alexandra B. Graf. Curation of the genome annotation of Pichia pastoris (Komagataella phaffii) CBS7435 from gene level to protein function. *FEMS Yeast Research*, 16(6), 07 2016.

[124] Ielka van der Sluis, Lennart Kloppenburg, and Gisela Redeker. PAT workbench: Annotation and evaluation of text and pictures in multimodal instructions. In *Proceedings of the Workshop on Language Technology Resources and Tools for Digital Humanities (LT4DH)*, pages 131–139, Osaka, Japan, December 2016. The COLING 2016 Organizing Committee.

[125] Maarten van Gompel and Martin Reynaert. FoLiA: A practical xml format for linguistic annotation - a descriptive and comparative study. *Computational Linguistics in the Netherlands Journal*, 3:63–81, 12/2013 2013.

[126] Cynthia Van Hee, Gilles Jacobs, Chris Emmery, Bart Desmet, Els Lefever, Ben Verhoeven, Guy De Pauw, Walter Daelemans, and Véronique Hoste. Automatic detection of cyberbullying in social media text. *PLOS ONE*, 13(10):1–22, 10 2018.

[127] Cynthia Van Hee, Els Lefever, Ben Verhoeven, Julie Mennes, Bart Desmet, Guy De Pauw, Walter Daelemans, and Veronique Hoste. Detection and fine-grained classification of cyberbullying events. In *Proceedings of the International Conference Recent Advances in Natural Language*

Processing, pages 672–680, Hissar, Bulgaria, September 2015. INCOMA Ltd. Shoumen, BULGARIA.

[128] Karin Verspoor, Antonio Jimeno Yepes, Lawrence Cavedon, Tara McIntosh, Asha Herten-Crabb, Zoë Thomas, and John-Paul Plazzer. Annotating the biomedical literature for the human variome. *Database*, 2013:bat019, 2013.

[129] Marta Vila, Manuel Bertran, M. Antònia Martí, and Horacio Rodríguez. Corpus annotation with paraphrase types: new annotation scheme and inter-annotator agreement measures. *Language Resources and Evaluation*, 49(1):77–105, Mar 2015.

[130] Veronika Vincze, György Szarvas, Richárd Farkas, György Móra, and János Csirik. The BioScope corpus: biomedical texts annotated for uncertainty, negation and their scopes. *BMC Bioinformatics*, 9(11):S9, Nov 2008.

[131] Géraldine Walther and Benoît Sagot. Speeding up corpus development for linguistic research: language documentation and acquisition in romansh tuatschin. In *Proceedings of the Joint SIGHUM Workshop on Computational Linguistics for Cultural Heritage, Social Sciences, Humanities and Literature*, pages 89–94, Vancouver, Canada, August 2017. Association for Computational Linguistics.

[132] Yajuan Wang, Steven R. Steinhubl, Chrisopher Defilippi, Kenney Ng, Shahram Ebadollahi, Walter F. Stewart, and Roy J. Byrd. Prescription extraction from clinical notes: Towards automating emr medication reconciliation. *AMIA Joint Summits on Translational Science proceedings. AMIA Joint Summits on Translational Science*, 2015:188–193, Mar 2015. 26306266[pmid].

[133] Hiroaki Yamada, Simone Teufel, and Takenobu Tokunaga. Annotation of argument structure in Japanese legal documents. In *Proceedings of the 4th Workshop on Argument Mining*, pages 22–31, Copenhagen, Denmark, September 2017. Association for Computational Linguistics.

[134] Hiroaki Yamada, Simone Teufel, and Takenobu Tokunaga. Building a corpus of legal argumentation in japanese judgement documents: towards structure-based summarisation. *Artificial Intelligence and Law*, 27(2):141–170, Jun 2019.

[135] Seid Muhie Yimam, Chris Biemann, Ljiljana Majnaric, Šefket Šabanović, and Andreas Holzinger. An adaptive annotation approach for biomedical entity and relation recognition. *Brain Informatics*, 3(3):157–168, Sep 2016.

[136] Seid Muhie Yimam, Iryna Gurevych, Richard Eckart de Castilho, and Chris Biemann. WebAnno: A flexible, web-based and visually supported system for distributed annotations. In *Proceedings of the 51st*

Annual Meeting of the Association for Computational Linguistics: System Demonstrations, pages 1–6, Sofia, Bulgaria, August 2013. Association for Computational Linguistics.

[137] Seid Muhie Yimam, Steffen Remus, Alexander Panchenko, Andreas Holzinger, and Chris Biemann. Entity-centric information access with human in the loop for the biomedical domain. In *Proceedings of the Biomedical NLP Workshop associated with RANLP 2017*, pages 42–48. INCOMA Ltd., 2017.

[138] Wajdi Zaghouani, Nizar Habash, Ossama Obeid, Behrang Mohit, Houda Bouamor, and Kemal Oflazer. Building an Arabic machine translation post-edited corpus: Guidelines and annotation. In *Proceedings of the Tenth International Conference on Language Resources and Evaluation (LREC 2016)*, pages 1869–1876, Portorož, Slovenia, May 2016. European Language Resources Association (ELRA).

[139] Elina Zarisheva and Tatjana Scheffler. Dialog act annotation for twitter conversations. In *Proceedings of the 16th Annual Meeting of the Special Interest Group on Discourse and Dialogue*, pages 114–123, Prague, Czech Republic, September 2015. Association for Computational Linguistics.

[140] Elina Zarisheva and Tatjana Scheffler. Dialog act annotation for twitter conversations. In *Proceedings of the 16th Annual Meeting of the Special Interest Group on Discourse and Dialogue*, pages 114–123, Prague, Czech Republic, September 2015. Association for Computational Linguistics.

[141] Chrysoula Zerva, Riza Batista-Navarro, Philip Day, and Sophia Ananiadou. Using uncertainty to link and rank evidence from biomedical literature for model curation. *Bioinformatics*, 33(23):3784–3792, 07 2017.

[142] Li Zhou, Suzanne V. Blackley, Leigh Kowalski, Raymond Doan, Warren W. Acker, Adam B. Landman, Evgeni Kontrient, David Mack, Marie Meteer, David W. Bates, and Foster R. Goss. Analysis of Errors in Dictated Clinical Documents Assisted by Speech Recognition Software and Professional TranscriptionistsErrors in Clinical Documents Created Using Speech Recognition SoftwareErrors in Clinical Documents Created Using Speech Recognition Software. *JAMA Network Open*, 1(3):e180530–e180530, 07 2018.

[143] Éva Mújdricza-Maydt, Silvana Hartmann, Iryna Gurevych, and Anette Frank. Combining semantic annotation of word sense & semantic roles: A novel annotation scheme for verbnet roles on german language data. In Nicoletta Calzolari (Conference Chair), Khalid Choukri, Thierry Declerck, Sara Goggi, Marko Grobelnik, Bente Maegaard, Joseph Mariani, Helene Mazo, Asuncion Moreno, Jan Odijk, and Stelios Piperidis, editors, *Proceedings of the Tenth International Conference on Language Resources and Evaluation (LREC 2016)*, Paris, France, may 2016. European Language Resources Association (ELRA).
